# Phase Fraction Modulation Enhances Li^+^/Na^+^ Diffusion Disparity in Spent LiFePO_4_ Cathodes for Efficient Lithium Extraction from Brine

**DOI:** 10.1002/advs.75320

**Published:** 2026-04-16

**Authors:** Ruiqi Yin, Qihan Huang, Jiayu Hao, Guosheng Li, Guoxing Ren, Dongfu Liu, Zhongwei Zhao, Wenhua Xu

**Affiliations:** ^1^ School of Materials Science and Engineering Zhengzhou University Zhengzhou China; ^2^ Zhongyuan Critical Metals Laboratory Zhengzhou University Zhengzhou China; ^3^ State Key Laboratory of Critical Metals Beneficiation Metallurgy and Purification Zhengzhou China; ^4^ The Key Lab of Critical Metals Minerals Supernormal Enrichment and Extraction Ministry of Education Zhengzhou China; ^5^ School of Metallurgy and Environment Central South University Changsha China

**Keywords:** cascaded utilization, Li/Na separation, phase fraction, secondary‐treatment lithium‐containing brine, spent LiFePO_4_, urban mining

## Abstract

Traditional spent LiFePO_4_ (SLFP) recycling faces challenges such as high energy consumption, environmental pollution, and low profitability. A novel direct cascaded utilization strategy was proposed for SLFP, where phase fraction modulation in the SLFP lattice enabled efficient lithium extraction from secondary‐treated lithium‐containing brine. The approach precisely controlled LiFePO_4_ phase (x in Li*_x_* FePO_4_) by regulating the oxidant dosage within a pH‐maintained reversible phase transition framework. Moreover, the contraction of the crystal structure and the amplified disparity in Li^+^/Na^+^ diffusion energy barriers synergistically improve structure stability and ion selectivity. The Li^+^/Na^+^ diffusion energy barrier difference was up to 0.56 eV. During the (de)intercalation process, Li_0.19_FePO_4_ exhibited excellent structure stability and Li/Na separation capability. The dissolution loss rates of Fe and P atoms were only 0.27% and 0.58%. And the maximum Li^+^ recovery rate can reach 99% within 40 min. Furthermore, the economic and environmental analysis indicated that the new strategy yields 528% higher profit ($7.23 per kg of SLFP), 40.5% reduction in energy demand and 67.8% reduction in greenhouse gas (GHG) emissions compared to conventional pyrometallurgy. It bypasses the need for pre‐sorting SLFP, enhancing SLFP recycling efficiency and promoting high‐value use of lithium‐containing brine through “urban mining”.

## Graphical abstract

A direct cascaded utilization strategy for spent LiFePO_4_ batteries efficiently extracts lithium from brine, embodying the “urban mining” concept. Phase fraction modulation revealed its significant impact on local bond lengths of crystal structure and the Li/Na diffusion energy barrier, identifying the best material (Li_0.19_FePO_4_) for lithium extraction. This yields battery‐grade Li_3_PO_4_, establishing a sustainable, high‐profit framework for circular lithium recovery.

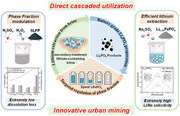

## 1 Introduction

Lithium‐ion batteries (LIBs) constitute a cornerstone of modern energy storage technology and have become a primary energy supply for portable electronics and electric vehicles [1, 2, 3]. The global demand for LIBs has been steadily increasing in recent years. By 2030, the total battery demand will reach 2800 GWh and is expected to continue growing, surpassing 9000 GWh by 2050 [4]. Among various types of LIBs, LiFePO_4_ batteries have gained significant market share due to their excellent cycle stability, high safety, and low production cost [5, 6, 7]. The rapid expansion of the energy storage market and electric vehicles over the past decade is expected to result in millions of tons of spent LIBs in the future [8, 9]. It is projected that spent LiFePO_4_ (SLFP) batteries will account for approximately 70% of the total spent LIBs [10]. On the one hand, SLFP batteries have significant resource value, containing considerable amounts of valuable metals such as lithium, iron, and copper. On the other hand, their hazardous components pose a significant threat to environmental contamination if disposed of improperly. Therefore, the effective recycling and disposal of SLFP batteries have become a critical challenge for the sustainable development of the industry.

The current methods for managing SLFP batteries primarily include three main ways: valuable metals recovery, regeneration and cascaded utilization. Conventional valuable metals recovery methods are broadly categorized into pyrometallurgical (Pyro) and hydrometallurgical (Hydro) processes. Pyro involves high‐temperature treatments to melt and separate metals from battery materials. While this approach is straightforward, it is plagued by high energy consumption and significant environmental emissions [11, 12]. Hydro is a process used to recover valuable materials from SLFP batteries through the use of aqueous chemistry, offering a more efficient and environmentally friendly alternative. However, this method suffers from low efficiency and lengthy processing steps and the generation of secondary pollutants [13, 14]. The regeneration method is to replenish lithium through chemical or physical methods to heal compositional and structural defects. But this method is still at the laboratory scale, suffering from difficulties in structural repair, complexity of the process, and limitations in material selection and reaction conditions [15, 16, 17, 18]. In comparison, cascaded utilization can bring more conveniently and efficiently into play the surplus value of SLFP. It is broadly used in fields with relatively low capacity requirements, including power tools, low‐speed electric vehicles, etc. [19, 20]. However, the uneven capacity decay of waste batteries will lead to problems such as difficulty in screening and reorganization and poor compatibility. Furthermore, a significant portion of decommissioned batteries also exist that cannot meet cascaded utilization requirements due to physical damage or safety hazards. Hence, it is required to develop a cascaded utilization method with wide adaptability to the source materials and compatibility with different quality grades to realize the efficient recycling of SLFP batteries.

Our group has proposed electrochemical intercalation/deintercalation (EID) method, which employs LiFePO_4_ cathode material as an excellent electrode active material for lithium extraction from salt lakes [21, 22, 23, 24, 25]. To ensure the kinetic performance and cyclic stability of the lithium extraction process, the practical working capacity of LFP electrodes is typically set at 20–30 mg•g^−1^ (approximately 50%–80% of the theoretical capacity) [26, 27]. Considering the capacity of general SLFP materials (approximately 80% of their original capacity) theoretically meets this operating range requirement, enabling them to be a potential lithium extraction material. However, using SLFP cathode material as the active electrode in conventional EID lithium extraction system necessitates a series of labor‐intensive physicochemical steps, including electrode disassembly, active material recovery, repulping, coating, and drying [28]. Furthermore, capacity uniformity is a critical requirement for electrode materials, as its absence leads to performance discrepancies, severe polarization, accelerated capacity decay, and structural damage, ultimately shortening the electrode's service life [29, 30]. However, retired cathode materials are inherently heterogeneous, particularly in their lithiation state and original formulation. Therefore, developing new strategies for the direct cascaded utilization of SLFP cathode material in lithium extraction is important. Given that the extraction process fundamentally depends on the reversible Fe^3^^+^/Fe^2^^+^ redox couple [31, 32, 33], we propose replacing the conventional electrochemical steps with chemical oxidation and reduction. Such a chemical‐driven approach can circumvent the tedious electrode preparation process and mitigate polarization issues arising from the non‐uniformity of SLFP materials.

Based on the above analysis, this paper proposes an efficient and environmentally friendly short‐process strategy for direct cascaded utilization of SLFP cathode material. This new strategy primarily consists of three key steps: modulating of phase fraction in SLFP to obtain Li*_x_* FePO_4_ (*x* adjusted from 1 to 0); lithium extraction from secondary‐treatment lithium‐containing brine (SLB) and natural salt lake brine (NLB) using Li*_x_* FePO_4_; the battery‐grade Li_3_PO_4_ product preparation. Concretely, for the phase fraction modulation process of SLFP cathode material, the pH of the solution was controlled using H_2_SO_4_ within the transition zone from the LiFePO_4_ to the FePO_4_ phase. And the H_2_O_2_ solution was added to realize the selective leaching of Li and to avoid the dissolution losses of Fe and P. For the lithium extraction from brine, utilizing Li*_x_* FePO_4_ and adding the reducing agent Na_2_SO_3_ to recover lithium ions efficiently. A deeper examination of how phase fraction influences the crystal structure and Li^+^/Na^+^ diffusion energy barrier, thereby affecting the structure stability and ion selectivity. The Li^+^ recovery rate reached 99%, and the dissolution loss rates of Fe and P atoms were only 0.27% and 0.58%. Subsequently, the above lithium deintercalation operations of SLFP material were repeated to achieve the enriched lithium solution. Finally, Na_3_PO_4_·12H_2_O was added to the oxidized leached lithium‐rich solution to obtain a battery‐grade Li_3_PO_4_ product with a purity of 98.90% in one step. In contrast to conventional pyrometallurgy, this method demonstrates superior performance by increasing profitability by 528%, cutting energy consumption by 40.5% and lowering greenhouse gas (GHG) emissions by 67.7%. This method can not only realize the direct cascaded utilization of SLFP cathode materials but also make the whole process more environmentally friendly, clean, and efficient.

## 2 Results and Discussion

### 2.1 Modulating Phase Fraction in Spent LiFePO_4_ Cathode

**FIGURE 1:**
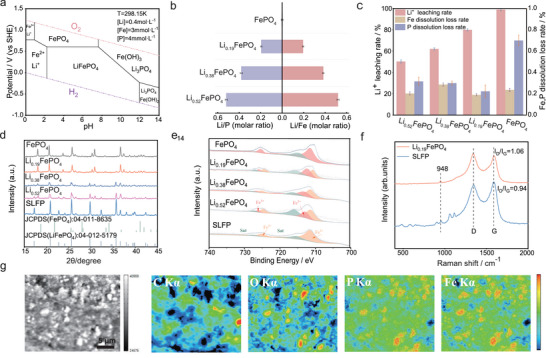
(a) E‐pH diagram of the Li─Fe─P─H_2_O system at 298 K. (b) Li/P and Li/Fe molar ratios of samples with different lithium contents. (c) Li^+^ leaching rate and dissolution loss rate for the corresponding Li*_x_* FePO_4_ samples. (d) XRD patterns. (e) XPS patterns. (f) Raman spectra of SLFP and Li_0.19_FePO_4_. (g) EPMA analysis of Li_0.19_FePO_4_.

To achieve targeted regulation of phase fraction in spent LiFePO_4_ while maintaining the material's structural stability, a theoretical analysis of potential reactions was conducted based on the solution composition. A Pourbaix diagram of the Li─Fe─P─H_2_O system is presented in Figure 1a. The thermodynamic data used to calculate the diagram was based on a temperature of 298 K. The corresponding thermodynamic data are listed in Tables S1 and S2. As shown in Figure 1a, the direct transformation of LiFePO_4_ to FePO_4_ required controlling the pH of the system between 2.2 and 7.6 and maintaining a potential higher than 0.6 V to achieve selective lithium removal. Based on the operability and controllability of the experiment, the pH was maintained between 3 and 4. Furthermore, H_2_O_2_ was chosen as a strong oxidant to enhance the oxidation potential of the system to ensure the efficient oxidation of Fe^2^^+^ to Fe^3^^+^. Thus, the H_2_SO_4_‐H_2_O_2_ system was chosen to leach residual lithium from SLFP materials. According to the stoichiometric ratios of the chemical reaction equation, the phase fraction of Li*_x_* FePO_4_ can be systematically tuned by the precise control of the (H_2_O_2_)/n(LiFePO_4_) ratio. As shown in Figure 1b,c, incrementally increasing this ratio enabled the gradient deintercalation of Li^+^. Concretely, the Li_0.52_FePO_4_, Li_0.38_FePO_4_, Li_0.19_FePO_4_, and FePO_4_ were obtained by adjusting the molar ratio of n(H_2_O_2_)/n(LiFePO_4_). As the amount of H_2_O_2_ added increased, the leaching rate of Li^+^ rose from 50.37% to 98.11%, which was consistent with the Li content in the solid samples obtained. The dissolution loss rates of Fe and P were below 0.29% and 0.7%, respectively, demonstrating that the selective leaching of Li was feasible. The X‐ray diffraction (XRD) phase analysis showed that the diffraction peaks of SLFP were attributable to the LiFePO_4_ (JCPDS No. 04‐012‐5179) phase of the orthorhombic crystal system (Figure 1d). With the increase of H_2_O_2_ addition, the samples transitioned from the LiFePO_4_ lithium‐rich phase to the FePO_4_ (JCPDS No. 04‐011‐8635) lithium‐poor phase. The X‐ray photoelectron spectroscopy (XPS) was employed to investigate the valence changes of Fe on the specimen surfaces (Figure 1e). The peaks observed at 711.1 and 724.7 eV were attributed to the Fe^2+^ species. The peaks associated with Fe^2+^ species appeared in the Fe 2p spectrum of SLFP. The peaks observed at 712.8 and 726.1 eV were ascribed to Fe^3+^ species [34]. The lack of Li^+^ leads to a charge imbalance, and some of the Fe^2+^ are oxidized to Fe^3+^. With the increase in H_2_O_2_ addition, the major binding energy peaks shifted to higher binding energies, and the area of the peaks related to Fe^2+^ decreased. This indicated that the Fe^2+^ in SLFP was partially oxidized to Fe^3+^, which is consistent with the XRD.

Thereinto, the Li_0.19_FePO_4_ was selected for Raman (Figure 1f) and electron probe microanalysis (EPMA) (Figure 1g) characterization. In Raman spectroscopy, two broad peaks were located at 1350 and 1600 cm^−1^, which were attributed to the D and G bands, respectively. The I_D_/I_G_ value of Li_0.19_FePO_4_ (1.06) was higher than that of SLFP (0.94), suggesting that oxidative treatment would lead to a larger disorder and smaller conductivity of Li_0.19_FePO_4_. The peaks between 900 and 1200 cm^−1^ were attributed to the ν3 and ν1 vibrational modes of the P‐O bonds, and the vibrational intensity of Li_0.19_FePO_4_ was significantly lower than that of SLFP, suggesting that a very large portion of the FePO_4_ phase was formed in Li_0.19_FePO_4_ that was lithium‐deficient [35, 36]. The distinctive spectral band at 948 cm^−1^ for Li_0.19_FePO_4_ was owed to the symmetry of the PO_4_^3−^ structure [37], indicating that the oxidation treatment had not destroyed the structure of SLFP, but affected the symmetry of the structure due to the leaching of Li. The EPMA elemental face‐sweep analysis of Li_0.19_FePO_4_ showed a high degree of overlap in the spatial distribution of the Fe‐Kα and P‐Kα signals, confirming the structural stability of the iron phosphorus skeleton during the oxidation process (Figure 1g). Combining the above experimental results, it can be concluded that the oxidation of SLFP using the H_2_O_2_─H_2_SO_4_ system could leach Li^+^ in a gradient to obtain Li*_x_* FePO_4_.

### 2.2 The Direct Cascaded Utilization of Li_x_FePO_4_ for Lithium Extraction from Brine

#### 2.2.1 The Li/Na Separation Performance of Li_x_FePO_4_ Obtained from SLFP

**FIGURE 2:**
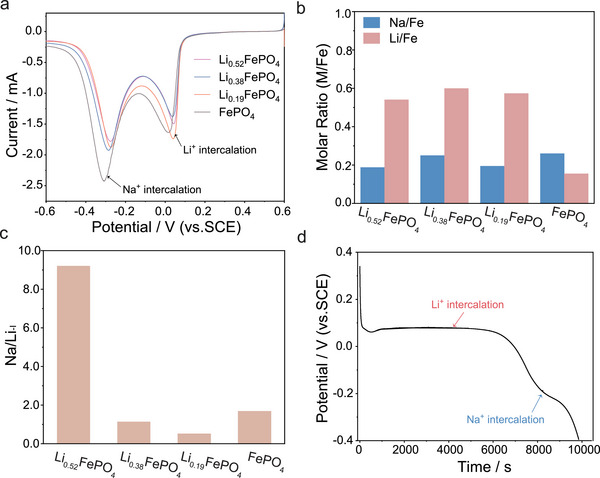
(a) LSV curves of Li*_x_* FePO_4_ materials. (b) Na/Fe ratio and Li/Fe ratio of Li*_x_* FePO_4_ electrodes after LSV test. (c) Na/Li_‐I_ of Li*_x_* FePO_4_ electrodes after LSV test. (d) CP curve of Li_0.19_FePO_4_ electrode at ‐0.4–0.4 V.

The Li/Na separation performance of Li*_x_* FePO_4_ obtained from SLFP has been investigated by means of electrochemical testing. In Figure 2a, the linear sweep voltammetry (LSV) results showed that the Li^+^ and Na^+^ intercalation characteristic peaks were located around 0.15 and ‐0.3 V, respectively. The Li_0.19_FePO_4_ displayed larger Li^+^ intercalation peak area and smaller Na^+^ intercalation peak area, which indicated the high Li^+^ selectivity of the material. Moreover, the FePO_4_ phase exhibited the largest peak area attributed to Na^+^ intercalation. It indicates that the complete removal of residual lithium from SLFP cathode materials is unfavorable for the Li/Na separation, which was in line with literature reports [38]. The compositions of the above reacted LSV electrodes were quantitatively analyzed using inductively coupled plasma (ICP), and the results are shown in Table S3 and Figure 2b,c, where Li_‐I_ refers to the reaction‐inserted lithium. Among them, Li_0.19_FePO_4_ exhibited the lowest Na/Li_‐I_ molar ratio of only 0.51. More than that, during the Li^+^ deintercalation process, the dissolution loss of Fe and P of FePO_4_ was higher than that of Li_0.19_FePO_4_ (Figure 1c). The inconsistency between the dissolution losses of Fe and P could be attributed to the fact that Fe would be partially hydrolyzed, while P would be fully dissolved. After comprehensive comparison, the Li_0.19_FePO_4_ material not only had excellent lithium intercalation ability but also showed excellent Li/Na separation performance. The chronopotentiometry (CP) test was further carried out to explore the separation performance of Li_0.19_FePO_4_ in the mixed solution (Figure 2d). The Li_0.19_FePO_4_ electrode showed a clear intercalation platform for Li^+^ and Na^+^ ions in the voltage range of ‐0.4–0.4 V. Accordingly, subsequent experiments may control the potential during the reaction to avoid the intercalation of sodium ions. To sum up, the Li_0.19_FePO_4_ is preferably selected for lithium extraction due to its excellent ion selectivity.

**FIGURE 3:**
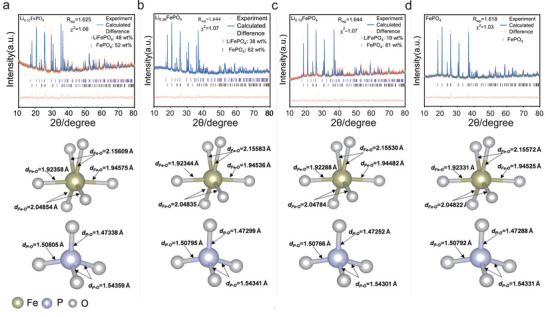
The XRD Rietveld refinement, local geometries, bond length in FeO_6_ octahedra and PO_4_ tetrahedron spectra of (a) Li_0.52_FePO_4_, (b) Li_0.38_FePO_4_, (c) Li_0.19_FePO_4_, (d) FePO_4_.

To further elucidate the intrinsic relationship between the compositional structure of SLFP derived Li*_x_* FePO_4_ materials and their Li/Na separation performance, the XRD Rietveld refinement was conducted to analyze the crystal structure, while density functional theory (DFT) calculations were performed to assess the diffusion barriers of Li^+^ and Na^+^ in various materials. As shown in Figure 3a–c, the bond lengths of Fe─O and P─O of the FePO_4_ phase in Li_0.52_FePO_4_, Li_0.38_FePO_4_, and Li_0.19_FePO_4_ gradually decrease. This phenomenon may be attributed to the oxidation of Fe^2+^ to Fe^3+^, which enhances the attractive force between Fe^3^^+^ and the surrounding oxygen atoms, resulting in a reduction of the Fe─O bond length. Furthermore, the covalent character of the P─O bond leads to a slight decrease in bond length during the delithiation process as the structure contracts [39, 40]. The shortening of bond lengths enhances covalent bonding, thereby contributing to greater structural stability. Upon reaching the fully delithiated state, a slight increase in bond lengths was observed (Figure 3d). This may be attributed to the formation of a metastable amorphous phase, where structural rearrangements lead to a minor rebound in bond lengths [41, 42]. These minor variations in bond lengths not only affect the local structural stability of the material but also indirectly influence the ion insertion energy barrier [40, 43].

**FIGURE 4:**
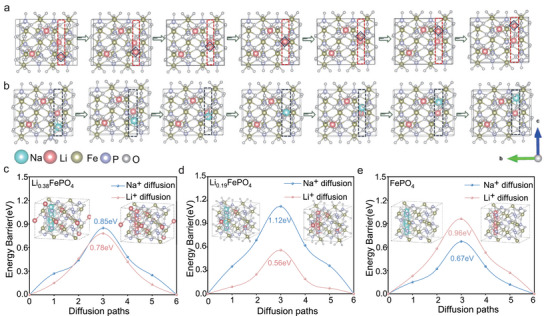
(a) Diffusion path of Li^+^ in Li_0.19_FePO_4_ structure. (b) Diffusion path of Na^+^ in Li_0.19_FePO_4_ structure. Simulated diffusion paths and energy barrier of Li^+^ and Na^+^ of the (c) Li_0.38_FePO_4_, (d) Li_0.19_FePO_4_, (e) FePO_4_.

Concretely, diffusion energy barriers for Li^+^ and Na^+^ in Li_0.38_FePO_4_, Li_0.19_FePO_4_, and FePO_4_ were computed using DFT, computational details see Text S1. Take Li_0.19_FePO_4_ as an example, the dynamic diffusion paths of Li^+^ and Na^+^ in Li_0.19_FePO_4_ were shown in Figures 4a,b. The diffusion energy barrier distributions for Li^+^ and Na^+^ were presented in Figure 4c–e. And the calculation results indicated that the energies required for Li^+^ diffusion in Li_0.38_FePO_4_, Li_0.19_FePO_4_, and FePO_4_ were measured at 0.78, 0.56, and 0.96 eV, while the corresponding energies for Na^+^ diffusion were 0.85, 1.12, and 0.67 eV, respectively. The Li_0.19_FePO_4_ exhibited the greatest difference in diffusion energy barriers for Li^+^ and Na^+^ (0.56 eV), suggesting optimal Li/Na separation capability. These theoretical calculations further explain the experimental findings presented in Figure 2. Additionally, they align with previous research indicating that partially filled lithium sites can raise the energy barrier for Na^+^ insertion [44, 45].

#### 2.2.2 The Lithium Extraction Performance of Li_0.19_FePO_4_

Although natural lithium minerals, as fundamental lithium resources, are crucial for the global new energy industry, the recycling and utilization of secondary‐treatment lithium‐containing resources are equally indispensable. With the surging global demand for lithium and escalating environmental pressures, recovering lithium from end‐of‐life batteries and industrial by‐products not only alleviates supply constraints on primary mineral deposits and mitigates ecological degradation associated with conventional mining practices but also enables resource circularity through the concept of “urban mining”.

**FIGURE 5:**
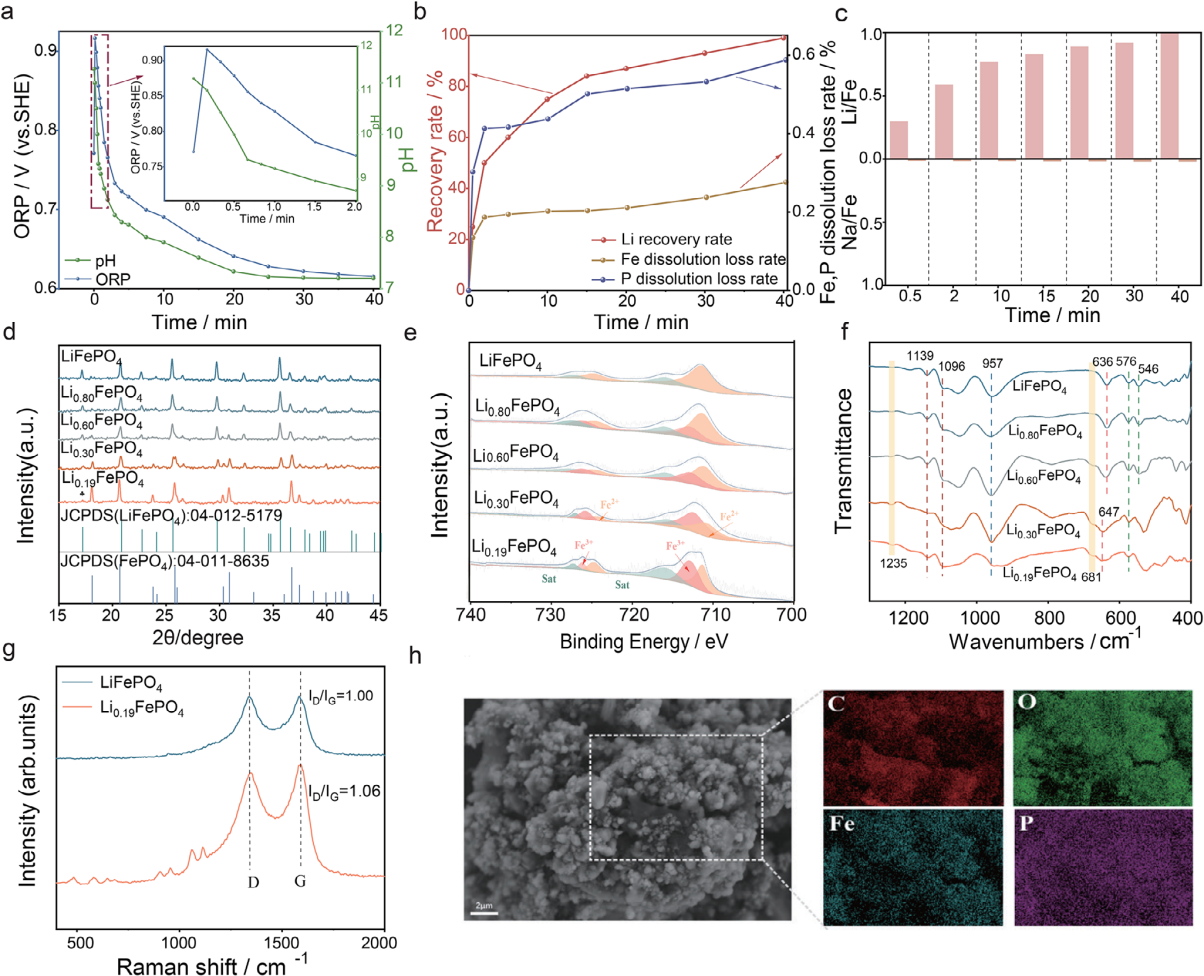
Lithium extraction from SLB‐spodumene. (a) OR P and pH value change curves. (b) Li^+^ recovery rate, Fe and P dissolution loss rates change curves during the whole lithium intercalation reaction. (c) Li/Fe and Na/Fe molar ratios of samples obtained at different reaction times. (d) XRD patterns. (e) XPS patterns. (f) FT‐IR patterns. (g) Raman spectra of Li_0.19_FePO_4_ (the initial state of the reaction) and LiFePO_4_ (the final state of the reaction). (h) SEM and EDS mapping of LiFePO_4_ (the final state of the reaction).

The subsequent content mainly delves into the lithium extraction processes from the secondary‐treated lithium‐containing brines derived from spodumene (abbreviated as SLB‐spodumene), lepidolite (abbreviated as SLB‐lepidolite), and end‐of‐life batteries (abbreviated as SLB‐batteries). In Figure 5, the lithium‐precipitated mother liquor generated from spodumene processing is selected as a lithium source, which contains 1.4‐2.2 g·L^−1^ of Li^+^ and 50–60 g·L^−1^ of Na^+^, and the pH is 11.3 [46, 47]. Noteworthily, if not treated, this mother liquor is directly discharged back to the salt lake, resulting in substantial resource waste [48]. Furthermore, in Section 2.2.1, the Li_0.19_FePO_4_ derived from SLFP exhibits efficient Li^+^ recovery property while maintaining high selectivity for Na^+^ and other ions. Therefore, in this section, Li_0.19_FePO_4_ was selected as an active material for lithium extraction. For the lithium extraction process, a substance with high reducing properties needs to be added. This substance must exhibit a lower reduction potential than that of the FePO_4_‐to‐LiFePO_4_ reduction reaction and enable rapid lithiation of Li_0.19_FePO_4_ at a lower temperature [34, 49]. Based on this, we propose to use Na_2_SO_3_ as a reducing agent and place Li_0.19_FePO_4_ to extract Li^+^ from the SLB.

Figure 5a shows the oxidation–reduction potential (ORP) of the solution and pH change curves of SLB‐spodumene during the lithium intercalation reaction. The pH rapidly decreased to 8.7 within 2 min, the pH was 7.2 at the end of the reaction. It is worth noting that the ORP value instantly increased from 0.77 V to 0.83 V (vs. SHE) within 1 min, which resulted from a one‐time addition of the reactants. Then, the reactants were evenly distributed, and Fe^3+^ was reduced, leading to weakened oxidizing capacity and decreased ORP values. According to the thermodynamic analysis detailed in Text S2, a potential difference exceeding 0.6 V was continuously maintained despite the pH decrease. This potential difference definitively demonstrated the thermodynamic spontaneity of the entire reaction. Furthermore, the stabilization of the ORP conclusively indicated the successful completion of the intercalation reaction. The concentration of Li^+^ at the initial stage of the reaction decreased rapidly, with the efficiency of Li^+^ recovery by the Li_0.19_FePO_4_ increasing significantly. Specifically, when the reaction time reached 40 min, the lithium concentration was 0.04 g·L^−1^, and the Li^+^ recovery rate reached 99%. Furthermore, the dissolution loss rates of Fe and P atoms during the reaction process were lower than 0.27% and 0.58% (Figure 5b). The Li/Fe and Na/Fe molar ratios during the reaction are shown in Figure 5c. The Li/Fe ratio increased sharply at the initial stage of the reaction, which was consistent with the rapid increase in the Li^+^ recovery rate. At the end of the reaction, the Li/Fe molar ratio reached 0.99, but the Na/Fe molar ratio was only 0.02, which further proved that Li_0.19_FePO_4_ had excellent ion selectivity. During this lithium intercalation reaction, the compositions and structures of solid samples taken at different times were analyzed. The XRD analysis reveals the phase transition pattern. In Figure 5d, the LiFePO_4_ and FePO_4_ phases coexisted in Li_0.19_FePO_4_, and the Li^+^ was supplemented chemically during lithium intercalation reaction. At the end of reaction, the final product was in a uniform LiFePO_4_ phase. In XPS spectra (Figure 5e), the peaks at 711.1 eV (2p_3/2_) and 724.7 eV (2p_1/2_) were attributed to Fe^2+^, and the peaks at 712.8 eV (2p_3/2_) and 726.1 eV (2p_1/2_) were ascribed to Fe^3+^ [50]. As the reaction proceeded, the Fe^2+^ content gradually increased, indicating that Li^+^ ions were gradually inserted into the crystal structure. The complete disappearance of the Fe^3+^ signaled at the end of the reaction, which further confirmed that Li_0.19_FePO_4_ had been completely converted into LiFePO_4_. The Fourier transform infrared spectroscopy (FT‐IR) and Raman analytical characterizations were used to further investigate the structure of the molecule. In Figure 5f, the peaks at 1235 and 681.13 cm^−1^ were attributed to the bending vibration of PO_4_^3−^ in FePO_4_. With the intercalation of Li^+^ ions, the diffraction peaks gradually weakened and finally the characteristic peaks disappeared, suggesting that Li_0.19_FePO_4_ was completely converted to LiFePO_4_ [10]. The peaks at 576 and 546 cm^−1^ were classified as bending modes (v2+v4) of PO_4_^3−^ in LiFePO_4_, where the peak at 546 cm^−1^ occurred at higher lithium intercalation, which agreed with the experimental results [51]. The peaks at 636 and 647 cm^−1^ were attributed to the LiFePO_4_, with the peak at 647 cm^−1^ dominating when the lithium content was low. The stretching vibration peak of the P‐O bond in the LiFePO_4_ without Fe─Li antisite defects was at 957 cm^−1^, indicating that no defects were generated during the extraction process [52]. The peaks at 1139 and 1096 cm^−1^ were attributed to the triplet state of the antisymmetric stretching mode v3 of PO_4_^3−^ [53], whose frequency remained constant.

The Raman spectra of Li_0.19_FePO_4_ (the initial state of the reaction) and LiFePO_4_ (the final state of the reaction) are shown in Figure 5g. The I_D_/I_G_ value of LiFePO_4_ (1.00) was lower than that of Li_0.19_FePO_4_ (1.06), suggesting that the structure was efficiently restored by the supplementation of lithium [54]. The scanning electron microscopy (SEM) and energy dispersive spectroscopy (EDS) patterns of the LiFePO_4_ (the final state of the reaction) are shown in Figure 5h. The morphology and particle size of the LiFePO_4_ remained essentially unchanged after cycling, and elements such as Fe and P were uniformly distributed in the material, suggesting that the structure of the regenerated material was still intact. Furthermore, standard coin cells were assembled using the recovered LiFePO_4_ to evaluate its practical viability. The comparative results definitively reveal the excellent electrochemical performance and stability of the regenerated material, specific content is shown in Figure S1. It should be noted that the SLFP used here represents manufacturing scrap with intact structures, making this strategy highly efficient for directly recycling manufacturing scraps.

**FIGURE 6:**
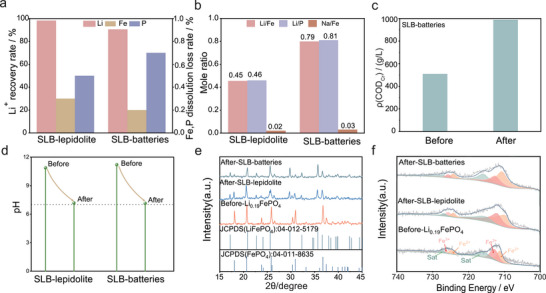
Lithium extraction from SLB‐lepidolite and SLB‐batteries. (a) Li^+^ recovery rate, Fe and P dissolution loss rates. (b) The molar ratios of Li/Fe, Li/P, and Na/Fe in the samples after lithium extraction. (c,d) The change of COD value and pH value in the SLB‐batteries solution before and after the lithium extraction reaction. (e,f) XRD patterns and XPS patterns of samples after lithium extraction.

In Figure 6, the SLB‐lepidolite and SLB‐batteries were selected as lithium sources. The ionic concentrations of above solutions before and after the lithium extraction reaction are detailed in Tables S4 and S5. As shown in Figure 6a, the Li^+^ recovery rates of Li_0.19_FePO_4_ of SLB‐lepidolite and SLB‐batteries were 98.13% and 90.47%, respectively. Upon completion of lithium extraction, the dissolution loss rates of Fe and P were also relatively low, all being less than 0.9%. The post‐reactive solids were subjected to ICP analysis (Figure 6b), yielding the compositions Li_0.46_FePO_4_ and Li_0.81_FePO_4_, which correspond to the respective amounts of extracted Li^+^ ions. As illustrated in Figure 6c, the chemical oxygen demands (COD) of SLB‐batteries before and after lithium extraction reaction were 510.5 and 989.55 g·L^−1^, respectively. The significant increase in the COD value of the post‐reaction solution can be attributed to the excess Na_2_SO_3_ added during the reaction process, which reacts with oxygen. The pH of the solution was alkaline at the outset and became neutral following the reaction, in compliance with discharge standards in Figure 6d. The XRD characterization of the sample obtained after lithium extraction was conducted, and the results revealed the presence of both LiFePO_4_ and FePO_4_ phases in the samples (Figure 6e). Concurrently, XPS analysis confirmed the coexistence of Fe^3+^ and Fe^2+^ in the samples (Figure 6f). The SEM and EDS spectra of the samples are presented in Figures S2 and S3, further substantiating the structural integrity of the materials after lithium extraction reaction.

In addition to SLB, this strategy remains applicable for lithium extraction from NLB, such as Zabuye salt lake (abbreviated as NLB‐Zabuye). The ionic concentrations before and after the lithium extraction reaction are provided in Table S6. The Li^+^ recovery rate of 91.97% was achieved. And the characterization results of samples after lithium extraction are shown in Figure S4. These results showed that the Li_0.19_FePO_4_ derived from SLFP not only enables efficient lithium extraction from NLB but also retained significant structural stability throughout the extraction process. Overall, this direct cascaded utilization strategy for SLFP cathode material is highly versatile, applicable to more than one type of lithium‐containing solution.

**FIGURE 7:**
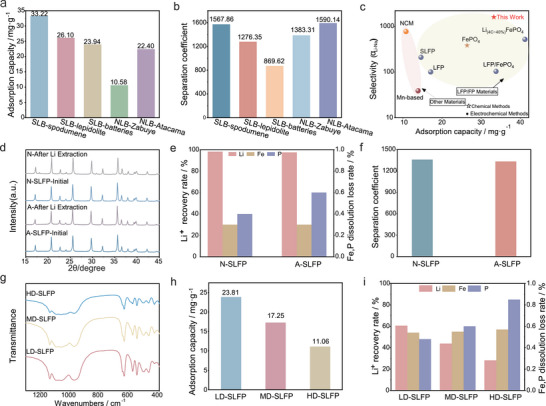
Universal lithium extraction performance and structural investigation. (a) Adsorption capacities across diverse lithium sources. (b) Separation coefficients for various feedstocks. (c) A comparison chart of the overall performance of this strategy with the latest reported literature. (d) XRD patterns showing structural unification of N‐SLFP and A‐SLFP samples at the initial state and after Li extraction. (e) Li^+^ recovery and impurity dissolution rates of N‐SLFP and A‐SLFP. (f) Separation coefficients of N‐SLFP and A‐SLFP. (g) FT‐IR spectra, (h) Adsorption capacities, (i) Li^+^ recovery and impurity dissolution rates of materials with varying states of health.

To further systematically evaluate the core competitiveness and broad universality of this strategy, the Li_0.19_FePO_4_ was tested across diverse brines, with the applied brine volume for each trial matched to the theoretical adsorption capacity of the material. The specific compositions of the above solutions are shown in Table S7. First, regarding the lithium extraction performance in multi‐source brine systems, the material exhibited exceptional results across distinct SLB and NLB. As depicted in Figure 7a, the adsorption capacity reached a peak of 33.22 mg•g^−1^ in high‐concentration SLB‐spodumene. The material maintained a capacity of 22.40 mg•g^−1^ even in the complex natural Atacama brine (abbreviated as NLB‐Atacama) with high magnesium content. Even in the more challenging NLB‐Zabuye, characterized by its complex carbonate matrix, a stable capacity of 10.58 mg•g^−1^ was achieved. It simultaneously achieved an outstanding lithium‐to‐sodium separation coefficient of 1590.14 (Figure 7b). Furthermore, Figure 7c illustrates a performance comparison between Li_0.19_FePO_4_ and recently reported similar lithium extraction materials. The detailed comparison data is provided in Table S8. Traditional manganese‐based adsorbents exhibit limited capacity and a selectivity with an *α*_Li‐Na_ value below 50. Electrochemical lithium extraction methods can achieve a relatively higher capacity. While electrochemical lithium extraction methods can achieve a relatively higher capacity, they typically display lower selectivity and require complex electrode preparation processes. By comparison, a highly competitive adsorption capacity approaching 33 mg•g^−1^, an exceptional α_Li‐Na_ separation factor exceeding 1000, and high chemical stability are simultaneously realized through this strategy.

Furthermore, this method demonstrated excellent process tolerance to the differences in spent raw materials and front‐end pre‐treatment processes. Commercial spent materials were pre‐treated using two different processes, organic solvent dissolution and high‐temperature calcination, and were respectively denoted as N‐SLFP and A‐SLFP for parallel experiments. The specific pre‐treatment flowcharts are detailed in Figures S5 and S6. Figure 7d displays the highly consistent XRD patterns of the final lithiated products (N‐After Li Extraction and A‐After Li Extraction). The complete compositional characterization is provided in Figure S7. Furthermore, regardless of the front‐end separation process used, these two spent materials ultimately achieved highly similar lithium extraction performance. Specifically, the Li^+^ recovery rates of both are greater than 98%, and the impurity dissolution is extremely low, as shown in Figure 7e. Simultaneously, the separation selectivity of both is also maintained at an extremely high level, with the corresponding results detailed in Figure 7f.

To evaluate the applicability of this strategy to authentic retired materials, the impact of cycling‐induced degradation was specifically investigated. Extensive literature reveals that capacity fading in SLFP batteries is primarily driven by the loss of lithium inventory rather than bulk structural collapse [55, 56]. Therefore, three authentic spent materials featuring gradient lithium deficiency were designated as LD‐SLFP, MD‐SLFP, and HD‐SLFP, with corresponding initial Li/Fe molar ratios of 0.90, 0.75, and 0.55, respectively. Although the basic olivine framework is generally preserved due to robust internal covalent bonds, extreme active lithium depletion inevitably exacerbates localized lattice distortions and lithium‐iron anti‐site defects. This structural deterioration is evidenced by Figure 7g, which displays significant peak broadening and intensity attenuation in the FT‐IR spectra as the retirement severity deepens. Figure S8 details the crystallographic, elemental, and morphological evolutions of these samples during the extraction process, confirming the successful phase transformation regardless of their initial degradation levels. However, as the lithium deficiency and coupled structural degradation deepen, the ultimate lithium adsorption capacity exhibits a clear gradient decline from 23.81 mg•g^−1^ for LD‐SLFP to 11.06 mg•g^−1^ for HD‐SLFP (Figure 7h). Concurrently, this intensified degradation significantly compromises the overall extraction efficiency, resulting in a substantial drop in the Li^+^ recovery rate accompanied by increased dissolution losses of structural Fe and P elements as shown in Figure 7i. Consequently, a more severe retirement degree directly leads to a lower adsorption capacity.

In summary, the lithium extraction capability of the proposed cascaded utilization strategy is not only influenced by the phase composition of the spent LFP but is also closely associated with the degradation degree of the spent batteries. Specifically, when the processed materials are manufacturing scraps, the strategy exhibits excellent lithium recovery efficiency. However, as the degradation level of the spent batteries increases, the lithium extraction performance of the recovered materials significantly declines due to the hindrance caused by thick SEI layers, aged binder encapsulation, and loss of lithium inventory [56, 57]. Consequently, the optimal candidates for this strategy are LFP manufacturing scraps, followed by battery materials with low degradation levels. The final lithium extraction performance essentially remains constrained by the coupled effects of the initial lithium inventory and the intrinsic structural integrity of the spent raw materials.

### 2.3 The Battery‐Grade Li_3_PO_4_ Product Preparation

**FIGURE 8:**
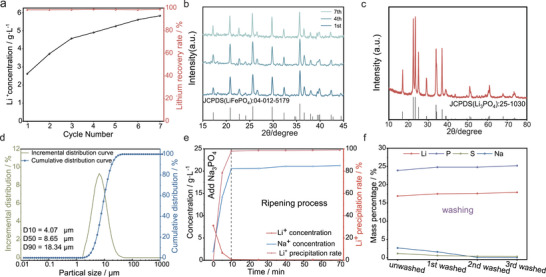
(a) The Li^+^ concentration of the purified lithium solution and the Li^+^ adsorption rate in seven cycles. (b) XRD patterns of samples after the 1st, 4th, and 7th cycles. (c) XRD patterns of the Li_3_PO_4_ product. (d) Laser particle size analysis of the Li_3_PO_4_ product. (e) The concentrations of Li^+^ and Na^+^ in the solution and the precipitation rate of Li^+^. (f) The concentrations of various ions in the Li_3_PO_4_ product during the rinsing process.

In Sections 2.1 and 2.2, targeted regulation of phase fraction in spent LiFePO_4_ is employed to enhance Li/Na separation in SLB and NLB. Lithium can be continuously extracted by repeating the lithium deintercalation/intercalation cycle. Noteworthily, after a single lithium leaching cycle, the resulting solution did not achieve the lithium concentration required for lithium phosphate precipitation (Li^+^ concentration > 5g·L^−1^). Therefore, this cycle was repeated seven times in this section. In Figure 8a, the Li^+^ recovery rates were stable at more than 98%, and the Li^+^ concentration of the purified lithium solution was elevated to 5.8 g·L^−1^ after sevenfold enrichment. The slight fluctuations observed during the enrichment process mainly result from unavoidable experimental volume variations, including routine sampling losses, filter cake entrainment, and reagent dilution. The detailed evolution of specific ion concentrations during each cycle is provided in Table S9. As shown in Figure S9, the lithium enrichment factor during cycling was all above 100, indicating that the system had excellent Li/Na separation performances. The lithium‐extraction active materials of the 1st, 4th, and 7th cycles were selected for XRD characterization (Figure 8b). The results revealed that all samples maintained the standard LiFePO_4_ olivine structure, confirming that the cycling process did not cause structural degradation of the material.

After Li^+^ ions enrichment, the Na_3_PO_4_·12H_2_O was added to prepare a high‐purity Li_3_PO_4_ product. In Figure 8c, the characteristic peaks of the product coincided with the characteristic peaks of standard Li_3_PO_4_ (PDF#25‐1030) [58] No impurity peaks were detected, and the high intensity of the characteristic peaks indicated the high crystallinity of the product. The laser particle size analysis (Figure 8d) showed that most of the Li_3_PO_4_ particles were in the range of 1–20 µm in size. Furthermore, during the Na_3_PO_4_ titration stage (0–10 min), the Li^+^ concentration in the solution dropped rapidly, and the Li^+^ precipitation rate rapidly rose to 97.9%. The Na^+^ concentration in the solution also grew to 20.5 g·L^−1^. During the ripening stage, the Na^+^ concentration remained almost constant, and the Li^+^ concentration gradually converged to 0 g·L^−1^, indicating that all Li^+^ in the solution had been precipitated (Figure 8e). After rinsing three times with the solid‐liquid ratio of 1:2, the mass fractions of Na and S were reduced to 0.07% and 0.31%, while the mass fractions of Li and P were 17.9% and 25.2%, respectively (Figure 8f). Above results indicated that the purity level of Li_3_PO_4_ was 98.90% (battery‐grade, YST637‐2022), which meets the requirements for the production of industrial‐grade LFP materials. The SEM showed that Li_3_PO_4_ consisted of a cluster of stacked spherical particles, with smooth surfaces and no adherents. The EDS surface scanning results showed that the P and O elements were uniformly distributed on the surface of the Li_3_PO_4_ in Figure S10.

### 2.4 Economic and Environmental Analysis

**FIGURE 9:**
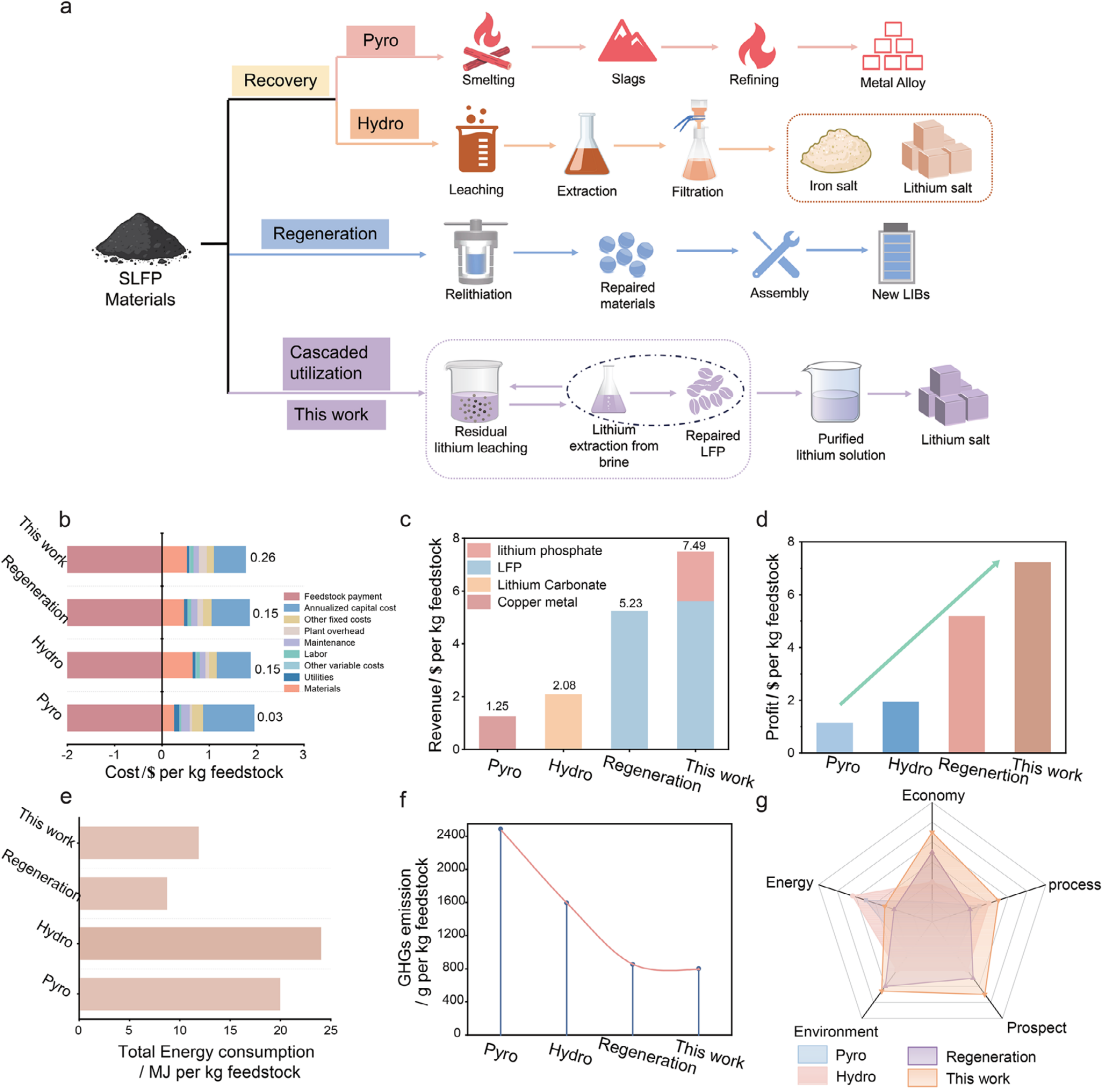
Comparison of economy and environment. (a) Simplified technical roadmap. (b) Cost analysis. (c) Revenue analysis. (d) Profit analysis. (e) Total energy consumption. (f) GHG emission. (g) Comprehensive evaluation chart.

In this section, we performed the economic and environmental analysis. Figure 9a illustrates the predominant methods currently employed for the recycling of SLFP, encompassing Pyro processes, Hydro techniques, direct regeneration approaches, and the methodologies involved in this work. Compared to other methods, the new strategy proposed in this paper can repeatedly utilize SLFP for the rapid and efficient recovery of Li^+^ ions from SLB and NLB. The detailed technological workflow is provided in Text S3. Concretely, the economic and environmental analysis of this new direct cascaded utilization strategy of spent LiFePO_4_ battery material for efficiently extracting lithium was conducted using the EverBatt2023 model.

Under a 10,000‐ton‐per‐year spent SLFP materials production scenario (Tables S10), we conducted the environmental and economic viability of SLFP recycling via Pyro, Hydro, regeneration, and cascaded utilization strategies. The detailed costs, revenues, and ultimate profits of each battery recycling technology are depicted in Figure 9b–d. See Tables S11–S13 for detailed information. indicating that the new direct cascaded utilization strategy yields a higher return compared to other methods. This is because it not only restores the SLFP structure but also produces lithium salt products. And the profit achieved in this work (7.23 $) represents a 5.28‐fold increase over the conventional Pyro strategy (1.15 $). It further demonstrated the stronger competitiveness of this new direct cascaded utilization strategy.

Energy consumption and environmental footprint are key indicators for evaluating the feasibility of battery recycling technologies (Table S14). The energy consumption of Pyro, Hydro, regeneration, and this work was 19.98, 24.04, 8.73, and 11.88 MJ/kg, respectively (Figure 9e). This new strategy eliminates high‐temperature requirements and minimizes chemical reagent usage, achieving a 40.5% reduction in energy demand compared to the conventional Pyro strategy. The new direct cascaded utilization strategy significantly reduces GHG emissions, accounting for only 32.2%, 50.3%, and 94.0% of those emitted by Pyro, Hydro and regeneration, respectively (Figure 9f). On a holistic level, the direct cascaded utilization strategy exhibits advantages such as high economic return, low energy consumption, cleanliness, and environmental protection (Figure 9g and Table S15). Thus, this strategy demonstrates significant potential for future applications.

## 3 Conclusion

The improper disposal of SLFP materials leads to irreversible loss of critical metal resources. However, current recycling technologies remain constrained by significant limitations, including high energy consumption, lengthy procedures, and low efficiency. This study proposes an efficient and environmentally friendly short‐process strategy. Modulation of phase fraction in SLFP revealed its significant impact on local bond lengths of the crystal structure and the Li/Na diffusion energy barrier, leading to the screening of Li_0.19_FePO_4_ as the best candidate material for subsequent lithium extraction from the secondary‐treated lithium‐containing brines. And the maximum Li^+^ recovery rate can reach 99% within 40 min. This strategy enables resource circularity through the concept of “urban mining”. This approach demonstrates remarkable improvements: 528% higher profitability, coupled with 40.5% reduction in energy consumption, and 67.7% decrease in GHG emissions compared with conventional pyro. The efficient and sustainable utilization of SLFP has been achieved through low‐energy consumption processes, enabling the secondary lithium liquid to be utilized with high added value, and achieving neutral emissions standards upon completion of the reaction. This study provides a novel industrial application approach for the recycling of waste battery materials, significantly broadening its scope of application.

## 4 Experimental Section

### 4.1 Entire Experimental Process

**FIGURE 10:**
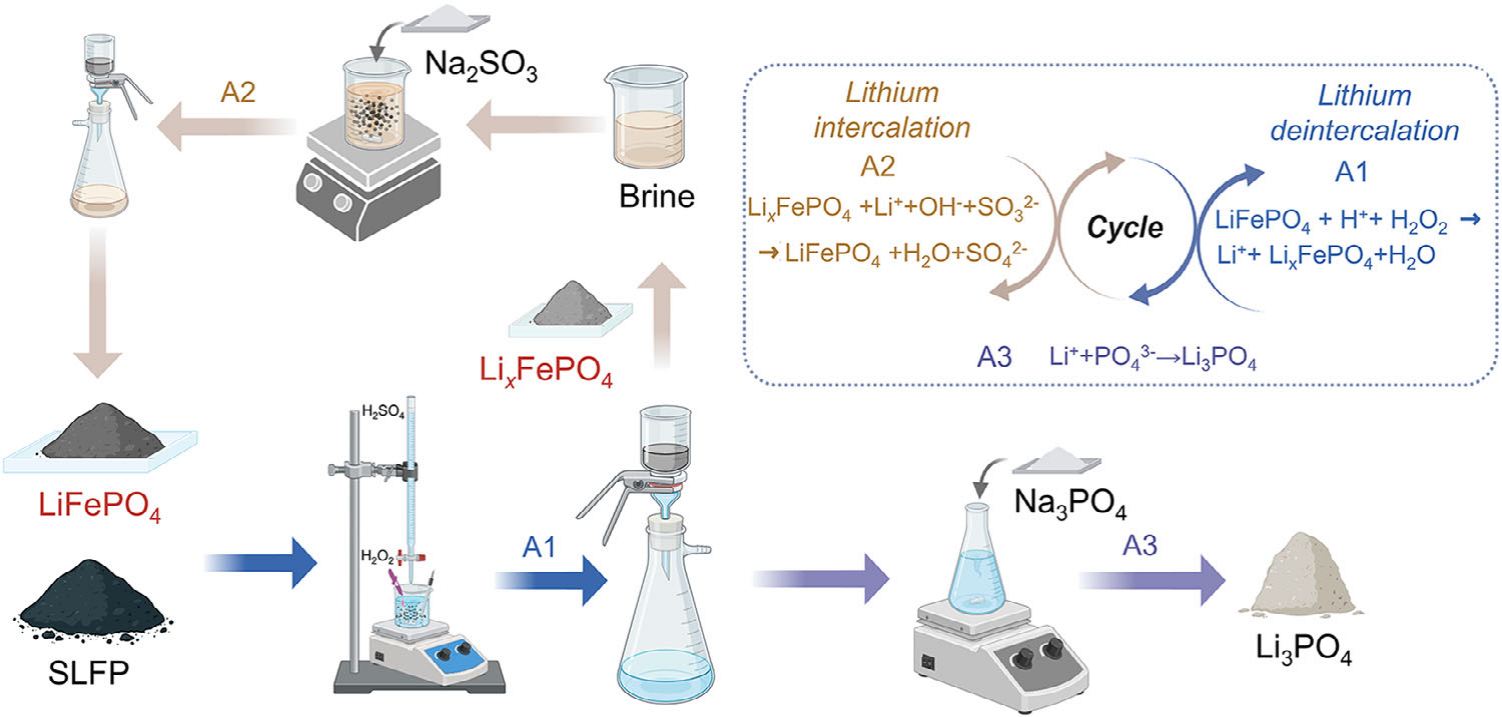
Flowchart of the whole process.

In Figure 10 the whole process consists of three parts: (1) targeted regulation of phase fraction in spent LiFePO_4_ cathode materials to obtain Li*_x_* FePO_4_; (2) direct cascaded utilization of delithiated lithium iron phosphate (Li*_x_* FePO_4_) for lithium extraction from SLB derived from spodumene, lepidolite, and end‐of‐life batteries and NLB. The specific compositions of the above solutions are shown in Table S7; (3) preparing of Li_3_PO_4_ from the obtained lithium‐rich leachate. Concretely, the phase fraction modulation process uses H_2_O_2_ and H_2_SO_4_ as oxidants and acidity regulators, respectively (2LiFePO_4_ + (1‐*x*) H_2_O_2_+ (1‐*x*) H_2_SO_4_ = 2Li*_x_* FePO_4_ + (1‐*x*) Li_2_SO_4_ + 2(1‐*x*) H_2_O). To maintain the stability of the LiFePO_4_ crystal structure, the pH value of the solution should be controlled between 3 and 4. The delithiated Li*_x_* FePO_4_ cathode materials were obtained by changing the amount of H_2_O_2_ and H_2_SO_4_. The Li*_x_* FePO_4_ can be used to directly extract lithium ions from the SLB and NLB. During the extraction process, Na_2_SO_3_ served as the reducing agent, and the reduction process follows the reaction equation: 2(1‐*x*) Li^+^ + 2Li*_x_* FePO_4_ + (1‐*x*) Na_2_SO_3_ +2 (1‐*x*) OH^−^ = 2LiFePO_4_ + (1‐*x*) Na_2_SO_4_ + (1‐*x*) H_2_O. The whole extraction process was carried out at 60 °C for 40 min. Lithium can be continuously extracted by repeating the lithium removal/adsorption cycle. Lithium leaching was carried out in the solution from which lithium was leached in the previous cycle as a way to achieve lithium enrichment. Subsequently, the lithium‐enriched solution can be treated with sodium phosphate to precipitate lithium phosphate products. This precipitation was achieved by adding sodium phosphate in a stoichiometric ratio, with the reaction occurring at 90°C for 60 min. Detailed materials, material characterizations, data analysis and electrochemical measurements are provided in the Supporting Information (Texts S4–S7).

## Conflicts of Interest

The authors declare no conflicts of interest.

## Supporting information




**Supporting File**: advs75320‐sup‐0001‐SuppMat.docx.

## Data Availability

The data that support the findings of this study are available from the corresponding author upon reasonable request.
