# Supplementary material for: Phase Fraction Modulation Enhances Li+/Na+ Diffusion Disparity in Spent LiFePO4 Cathodes for Efficient Lithium Extraction from Brine
Source: Adv Sci (Weinh). 2026 Apr 16;13(38):e75320. doi: 10.1002/advs.75320 (PMC13335503; doi:10.1002/advs.75320)
Supplement: Supplementary file 1 — Supporting File: advs75320‐sup‐0001‐SuppMat.docx. [file ADVS-13-e75320-s001.docx]

**Supporting Information**

**Phase Fraction Modulation Enhances Li^+^/Na^+^ Diffusion Disparity in Spent LiFePO_4_ Cathodes for Efficient Lithium Extraction from Brine**

Ruiqi Yin ^1, 2^, Qihan Huang ^1, 2^, Jiayu Hao ^2, 3, 4*^, Guosheng Li ^1, 2, 3, 4^, Guoxing Ren ^2, 3, 4^, Dongfu Liu ^2, 3, 4^, Zhongwei Zhao ^5^, Wenhua Xu ^2, 3, 4*^

^1^ School of Materials Science and Engineering, Zhengzhou University, Zhengzhou 450001, China

^2^ Zhongyuan Critical Metals Laboratory, Zhengzhou University, Zhengzhou 450001, China

^3^ State Key Laboratory of Critical Metals Beneficiation, Metallurgy and Purification, Zhengzhou, 450001, China

^4^ The Key Lab of Critical Metals Minerals Supernormal Enrichment and Extraction, Ministry of Education, Zhengzhou 450001, China

^5^ School of Metallurgy and Environment, Central South University, Changsha 410083, China

* Correspondence: Jiayu Hao ([haojiayu@zzu.edu.cn](mailto:haojiayu@zzu.edu.cn)) ORCiD: 0009-0009-2963-3294| Wenhua Xu ([xuwenhua@zzu.edu.cn](mailto:xuwenhua@zzu.edu.cn)) ORCiD: 0009-0006-2282-9428

**Contents**

[**Figure S1.** a) Galvanostatic charge-discharge profiles of SLFP and LiFePO_4_ after cycling at 0.1 C. b) Rate capability from 0.1 C to 5 C. c) Long-term cycling performance and Coulombic efficiency at 1 C for 200 cycles. 4](#_Toc223609432)

[**Figure S2.** SEM and EDS mapping of samples after lithium extraction from SLB-lepidolite 5](#_Toc223609433)

[**Figure S3.** SEM and EDS mapping of samples after lithium extraction from SLB-batteries 6](#_Toc223609434)

[**Figure S4.** a) The molar ratios of Li/Fe, Li/P and Na/Fe in the sample after lithium extraction from NLB-Zabuye. b,c) XRD patterns and XPS patterns of samples before and after lithium extraction from NLB-Zabuye 7](#_Toc223609435)

[**Figure S5.** Experimental steps of the organic solvent dissolution method (NMP Method) 8](#_Toc223609436)

[**Figure S6.** Experimental steps of the thermal treatment method 9](#_Toc223609437)

[**Figure S7.** Evolution of elemental mole ratios (Li/Fe, Li/P, and Na/Fe) for the a) N-SLFP and b) A-SLFP samples at the initial state and after Li extraction 10](#_Toc223609438)

[**Figure S8.** Crystallographic, elemental, and morphological evolutions of the SLFP materials with gradient degradation levels during the extraction process. a-c) XRD patterns, d-f) variations of elemental mole ratios (Li/Fe, Li/P, and Na/Fe), g-i) SEM images corresponding to the LD-SLFP, MD-SLFP, and HD-SLFP samples, respectively 11](#_Toc223609439)

[**Figure S9.** Lithium enrichment factor during cycles 12](#_Toc223609440)

[**Figure S10.** SEM image and EDS mapping images of Li_3_PO_4_ product 13](#_Toc223609441)

[**Table S1.** Thermodynamic data of components in the Li-Fe-P-H_2_O systems at 298.15K 14](#_Toc223609442)

[**Table S2.** E-pH formulas of equilibrium reaction in the Li-Fe-P-H_2_O systems at 298.15 K 15](#_Toc223609443)

[**Table S3.** ICP analysis of the materials after LSV 16](#_Toc223609444)

[**Table S4.** The composition of the SLB-lepidolite before/after lithium extraction reaction 17](#_Toc223609445)

[**Table S5.** The composition of SLB-batteries before/after lithium extraction reaction 18](#_Toc223609446)

[**Table S6.** The composition of NLB-Zabuye before/after lithium extraction reaction 19](#_Toc223609447)

[**Table S7.** The composition of SLB and NLB solutions 20](#_Toc223609448)

[**Table S8.** Comparison of lithium extraction performance between Li_0.19_FePO_4_ and representative materials reported in recent literature 21](#_Toc223609449)

[**Table S9.** Detailed evolution of ion concentrations during Step 1 and Step 2 processes over 7 cycles 22](#_Toc223609450)

[**Table S10.** Preprocessing throughput information 23](#_Toc223609451)

[**Table S11.** Basic and original data of techno-economic analysis 24](#_Toc223609452)

[**Table S12.** Recycling cost ($ per kg feedstock) of different battery recycling technologies 25](#_Toc223609453)

[**Table S13.** Recycling profit ($ per kg feedstock) of different battery recycling technologies 26](#_Toc223609454)

[**Table S14.** Life-cycle environmental impacts of different recycling methods 27](#_Toc223609455)

[**Table S15.** Comparison the technical and economic index of different methods 28](#_Toc223609456)

[**Text S1.** Computational details 29](#_Toc223609457)

[**Text S2.** Thermodynamic Calculation of Redox Potentials 30](#_Toc223609458)

[**Text S3.** Economic Evaluation Details 31](#_Toc223609459)

[**Text S4.** Materials 32](#_Toc223609460)

[**Text S5.** Material characterization 33](#_Toc223609461)

[**Text S6.** Data analysis 34](#_Toc223609462)

[**Text S7.** Electrochemical measurements 36](#_Toc223609463)

[**References** 37](#_Toc223609464)

To evaluate the practical electrochemical performance, standard coin cells were respectively assembled using the SLFP and the LiFePO_4_ material recovered after one complete delithiation-lithiation cycle. Figure S1a compares the galvanostatic charge-discharge profiles at 0.1 C within a voltage window of 2.5 to 4.3 V. The LiFePO_4_ after cycling exhibits a reversible discharge capacity of approximately 154 mAh⋅g^-1^, which is highly comparable to the 158 mAh⋅g^-1^ delivered by the original SLFP. Both materials display typically flat voltage plateaus, indicating preserved olivine structure and low electrochemical polarization. The rate performance from 0.1 C to 5 C is illustrated in Figure S1b, and long-term cycling stability at 1 C is depicted in figure 1c. These results further reveal the excellent rate capability and stability of the material. Specifically, the capacity retention reaches 91.6% after 200 cycles with coulombic efficiency remaining near 100%, matching the performance of the feedstock and validating the potential for a direct closed-loop process.


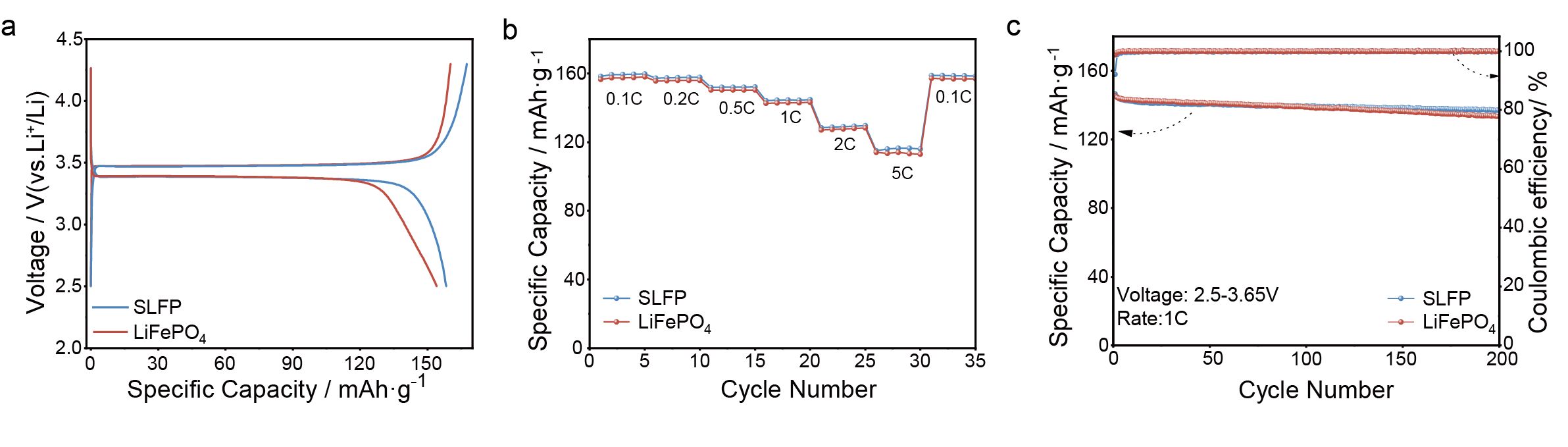


**Figure S1.** a) Galvanostatic charge-discharge profiles of SLFP and LiFePO_4_ after cycling at 0.1 C. b) Rate capability from 0.1 C to 5 C. c) Long-term cycling performance and Coulombic efficiency at 1 C for 200 cycles.


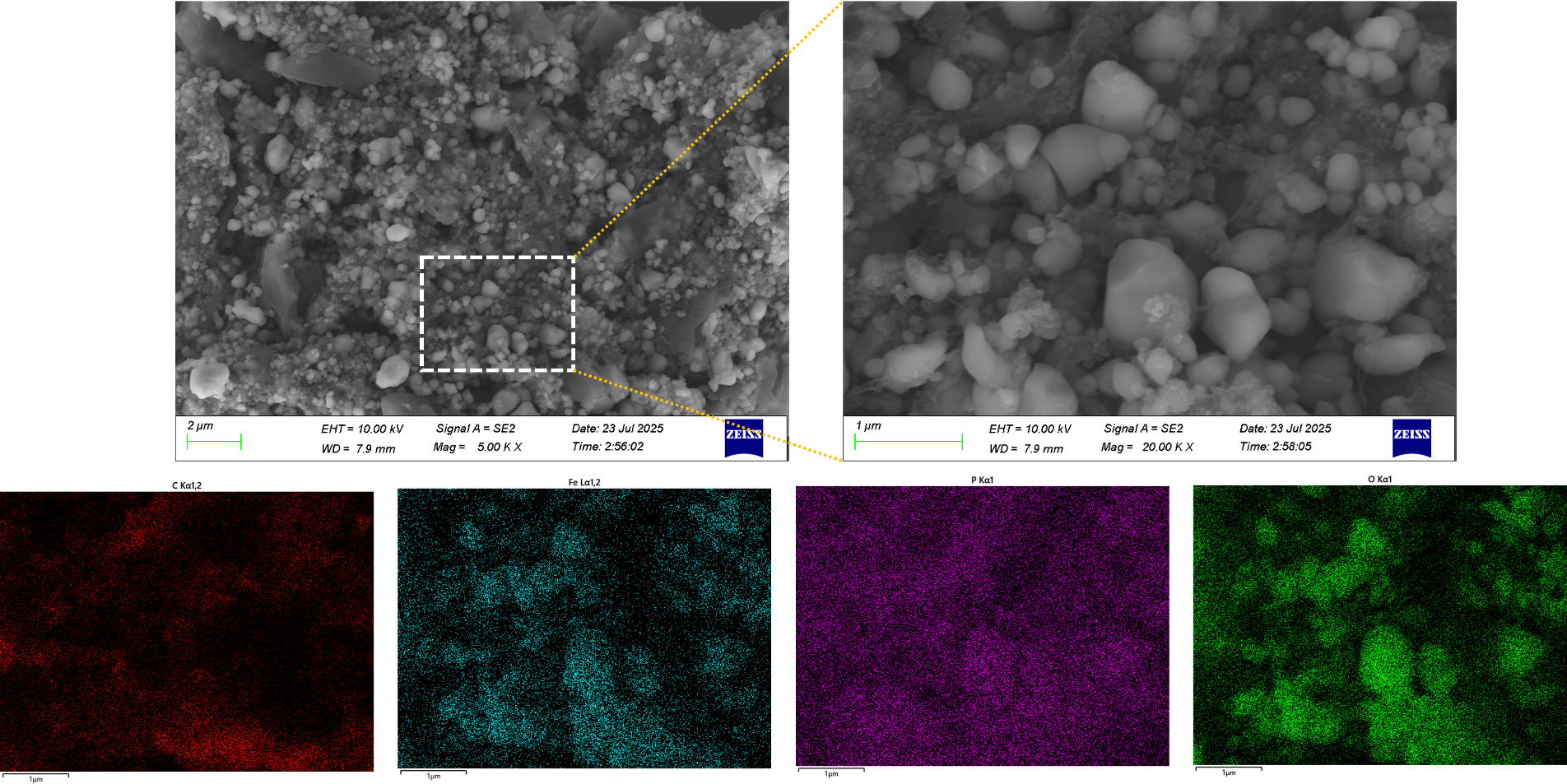


**Figure S2.** SEM and EDS mapping of samples after lithium extraction from SLB-lepidolite

#

**
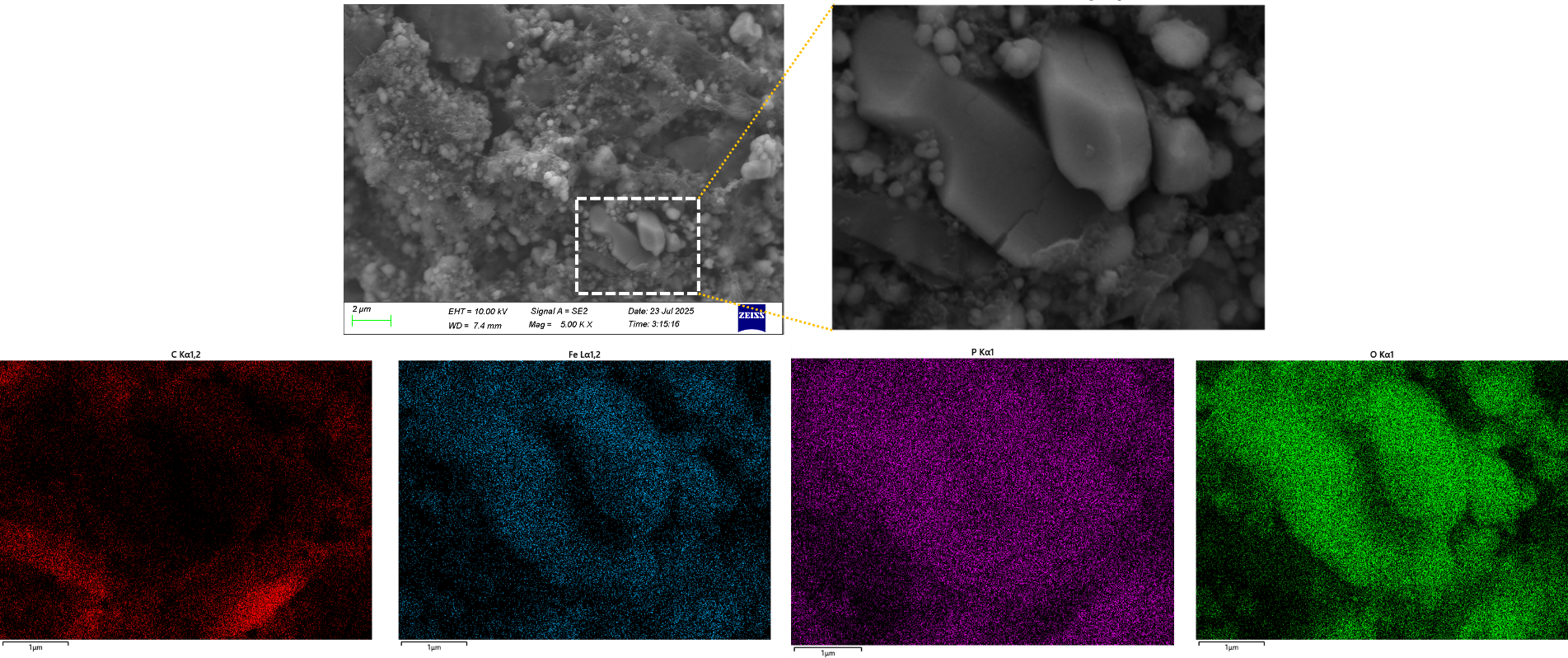
**

**Figure S3.** SEM and EDS mapping of samples after lithium extraction from SLB-batteries


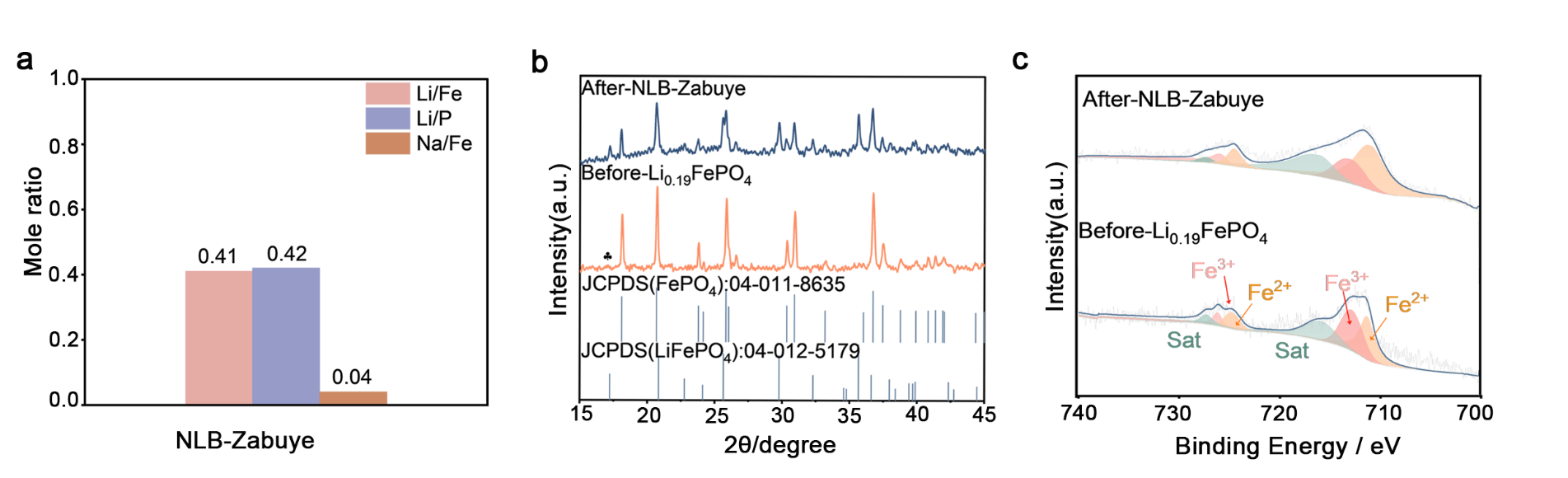


**Figure S4.** a) The molar ratios of Li/Fe, Li/P and Na/Fe in the sample after lithium extraction from NLB-Zabuye. b,c) XRD patterns and XPS patterns of samples before and after lithium extraction from NLB-Zabuye

Separation experiments were conducted on commercial spent LiFePO_4_ electrode sheets (supplied by Zhongneng Lithium Battery Technology Co., Ltd.). The specific experimental procedure is illustrated in Figure S5. Initially, 20 g of electrode fragments were placed in a beaker and immersed in dimethyl carbonate (DMC) for 10-15 min to remove residual electrolyte, followed by drying at 60 ℃. Subsequently, the cleaned electrode scraps were immersed in an N-methyl-2-pyrrolidone (NMP) solution. The mixture was heated to 80 ℃ in a water bath and stirred continuously for 60 min. During this process, the black powder gradually detached from the aluminum foil, turning the solution black. The resulting black slurry was subjected to vacuum filtration and washed repeatedly with anhydrous ethanol. The filter cake was then dried in a vacuum oven at 80 ℃ for 12 h. Finally, the dried material was gently ground in a mortar to obtain the final product, denoted as N-SLFP.


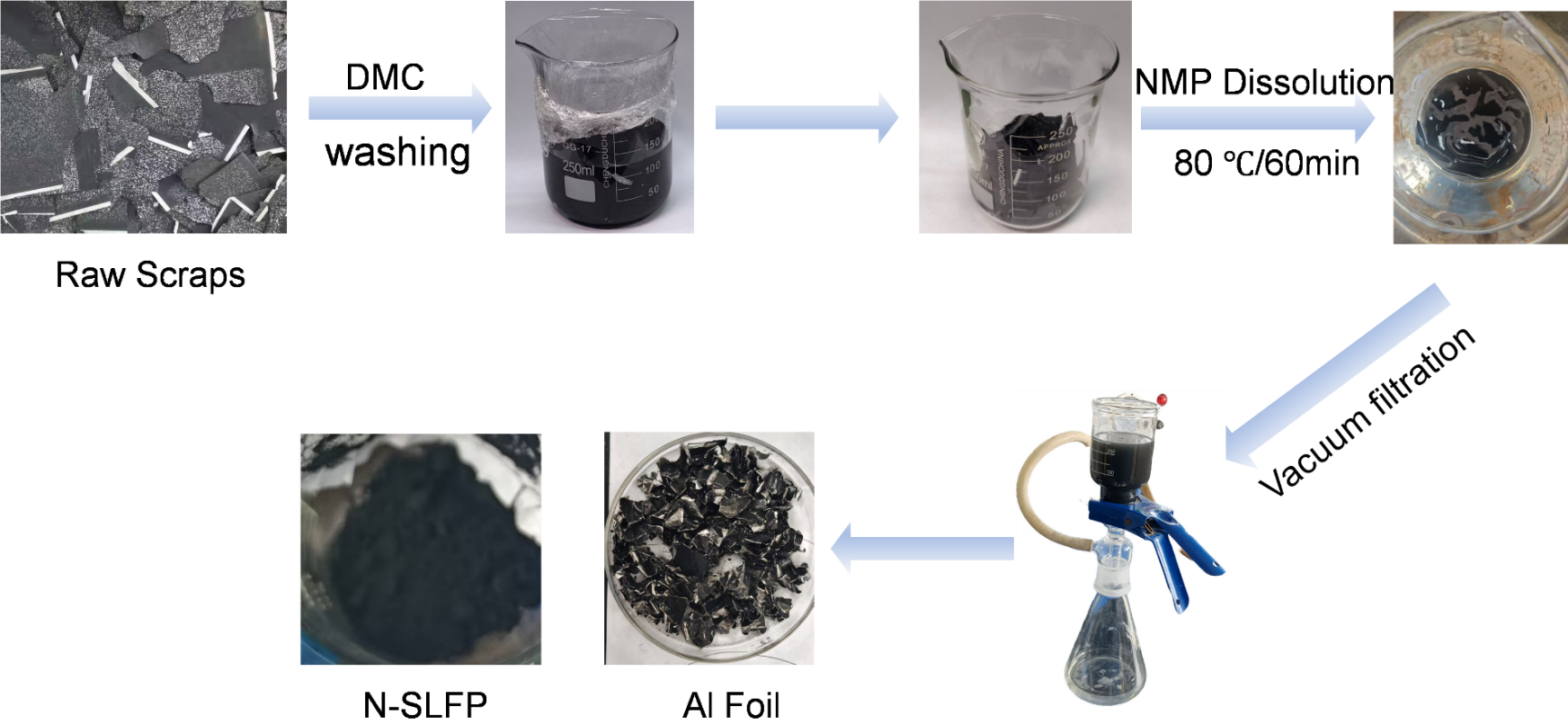


**Figure S5.** Experimental steps of the organic solvent dissolution method (NMP Method)

The specific experimental procedure is illustrated in Figure S6. The electrode sheets were cut into strips of appropriate dimensions and placed into a quartz boat. Prior to heating, the tube furnace was purged with Ar gas to eliminate air. The samples were then heated to 600 ℃ at a ramp rate of 5 ℃/min and calcined for 4 h. After the furnace naturally cooled to room temperature under the Ar atmosphere, the gas flow was terminated, and the samples were retrieved. The cathode powder, which had separated from the aluminum foil, was collected and ground to yield the final product, denoted as A-SLFP.


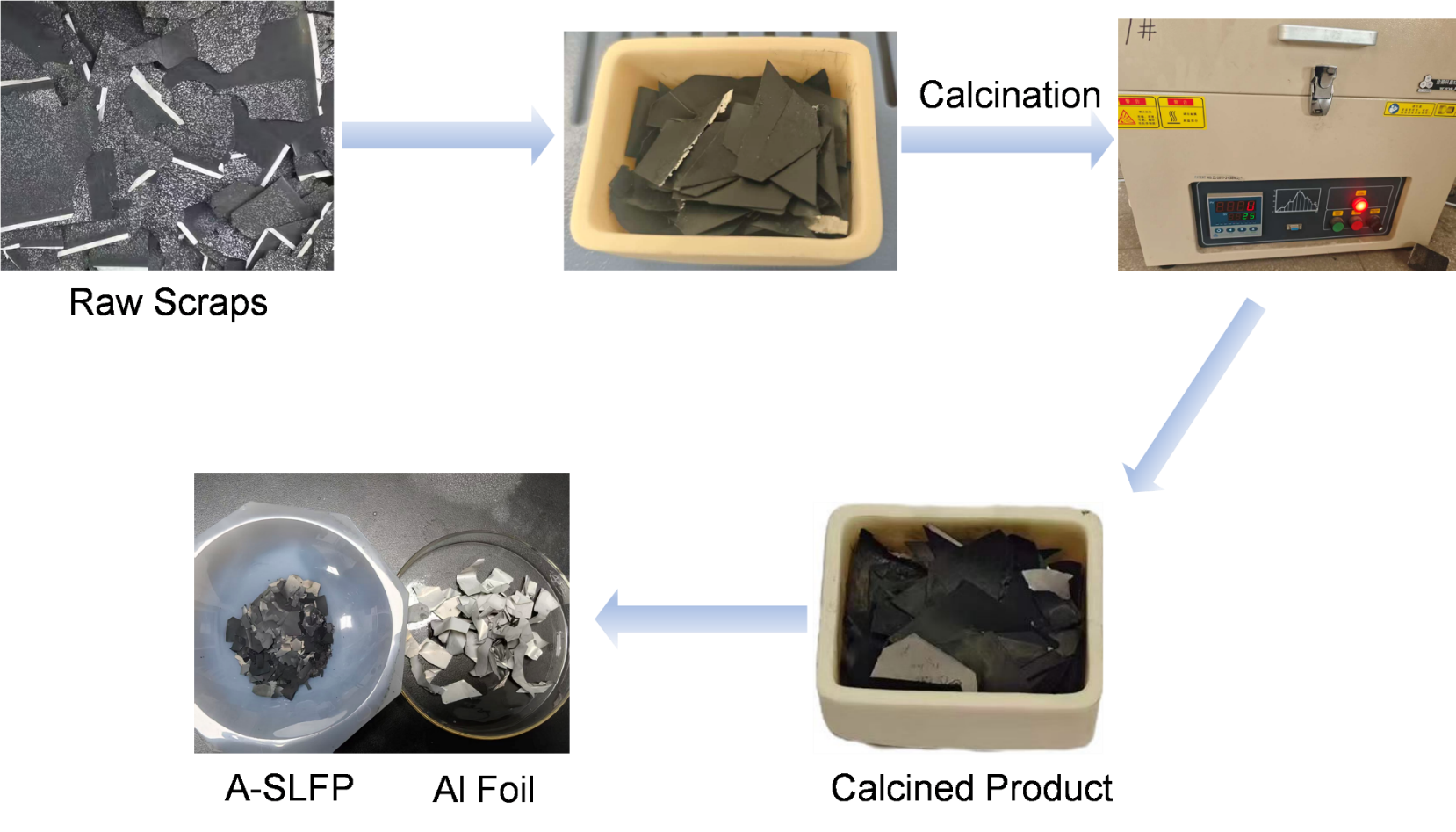


**Figure S6.** Experimental steps of the thermal treatment method


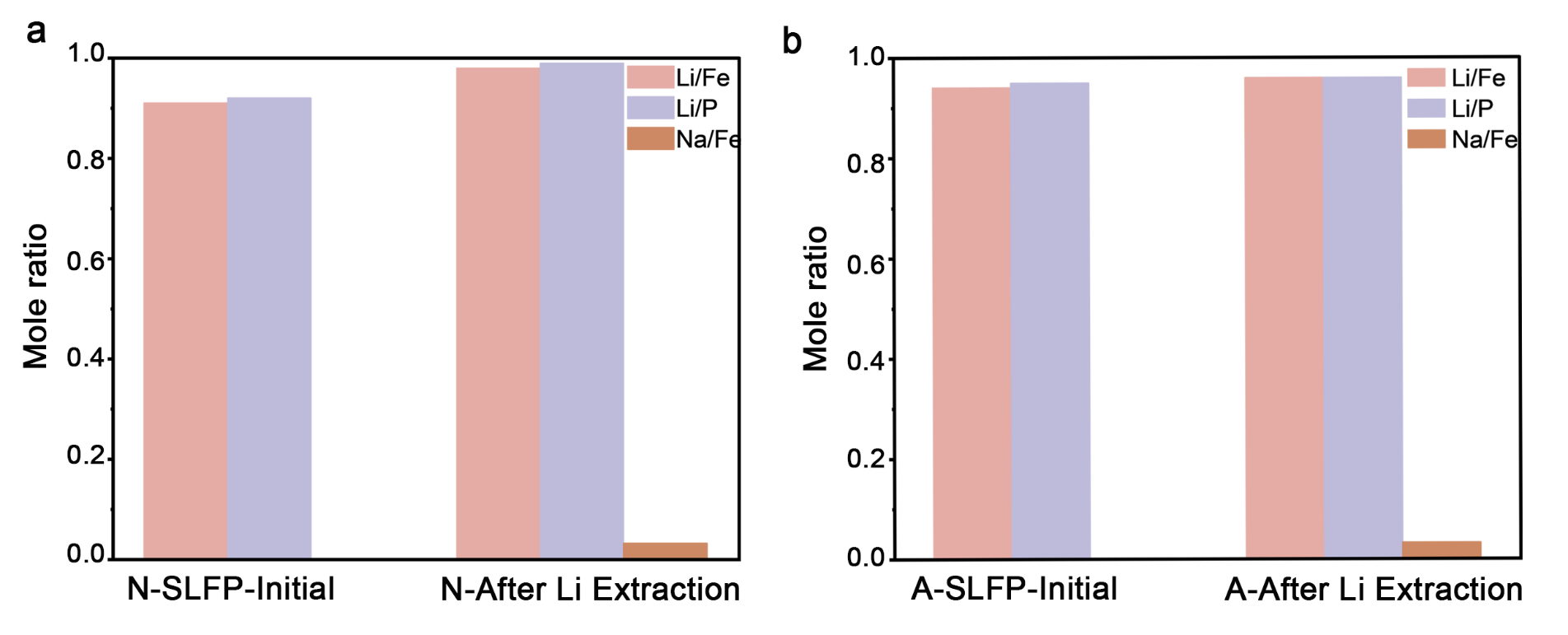


**Figure S7.** Evolution of elemental mole ratios (Li/Fe, Li/P, and Na/Fe) for the a) N-SLFP and b) A-SLFP samples at the initial state and after Li extraction


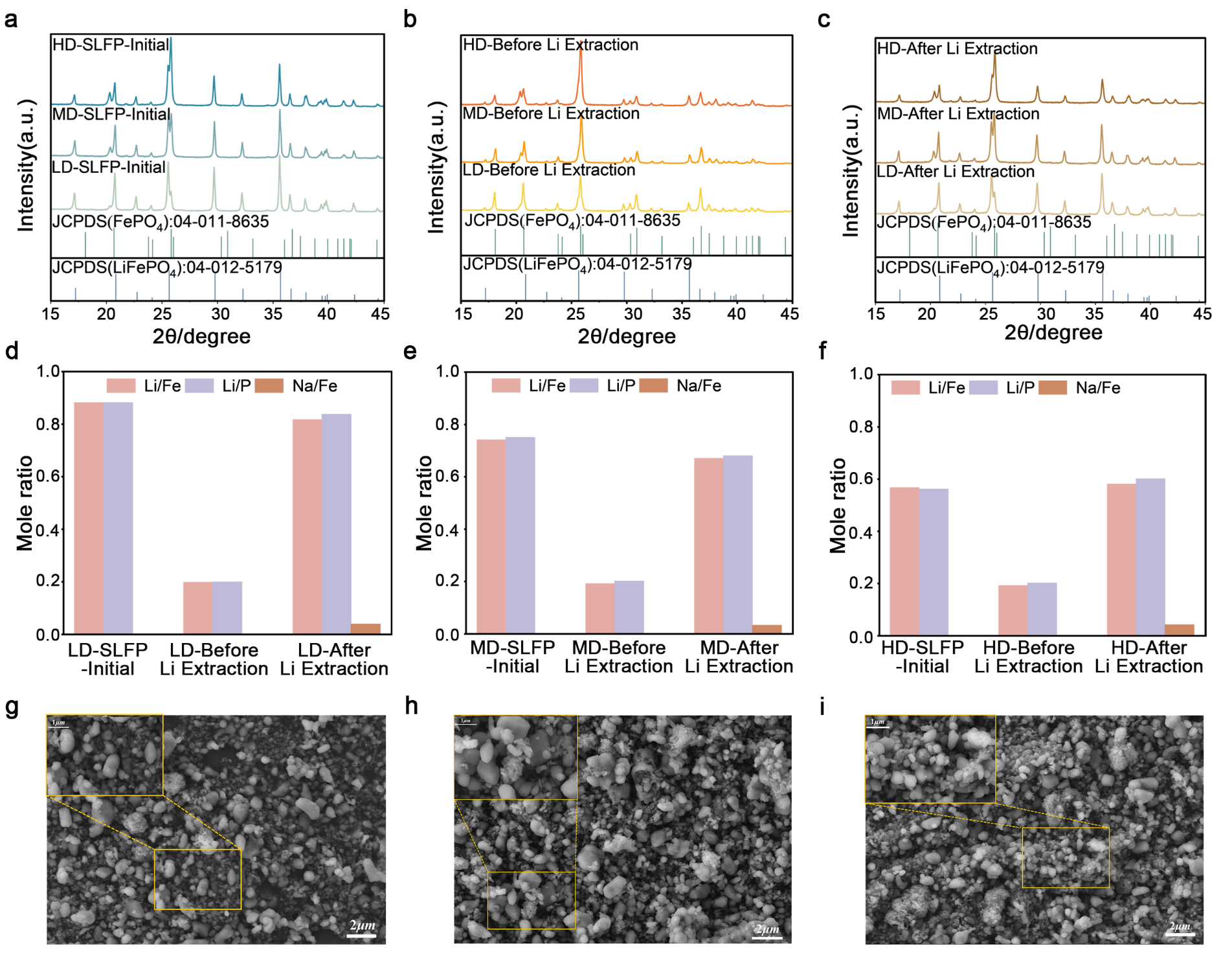


**Figure S8.** Crystallographic, elemental, and morphological evolutions of the SLFP materials with gradient degradation levels during the extraction process. a-c) XRD patterns, d-f) variations of elemental mole ratios (Li/Fe, Li/P, and Na/Fe), g-i) SEM images corresponding to the LD-SLFP, MD-SLFP, and HD-SLFP samples, respectively


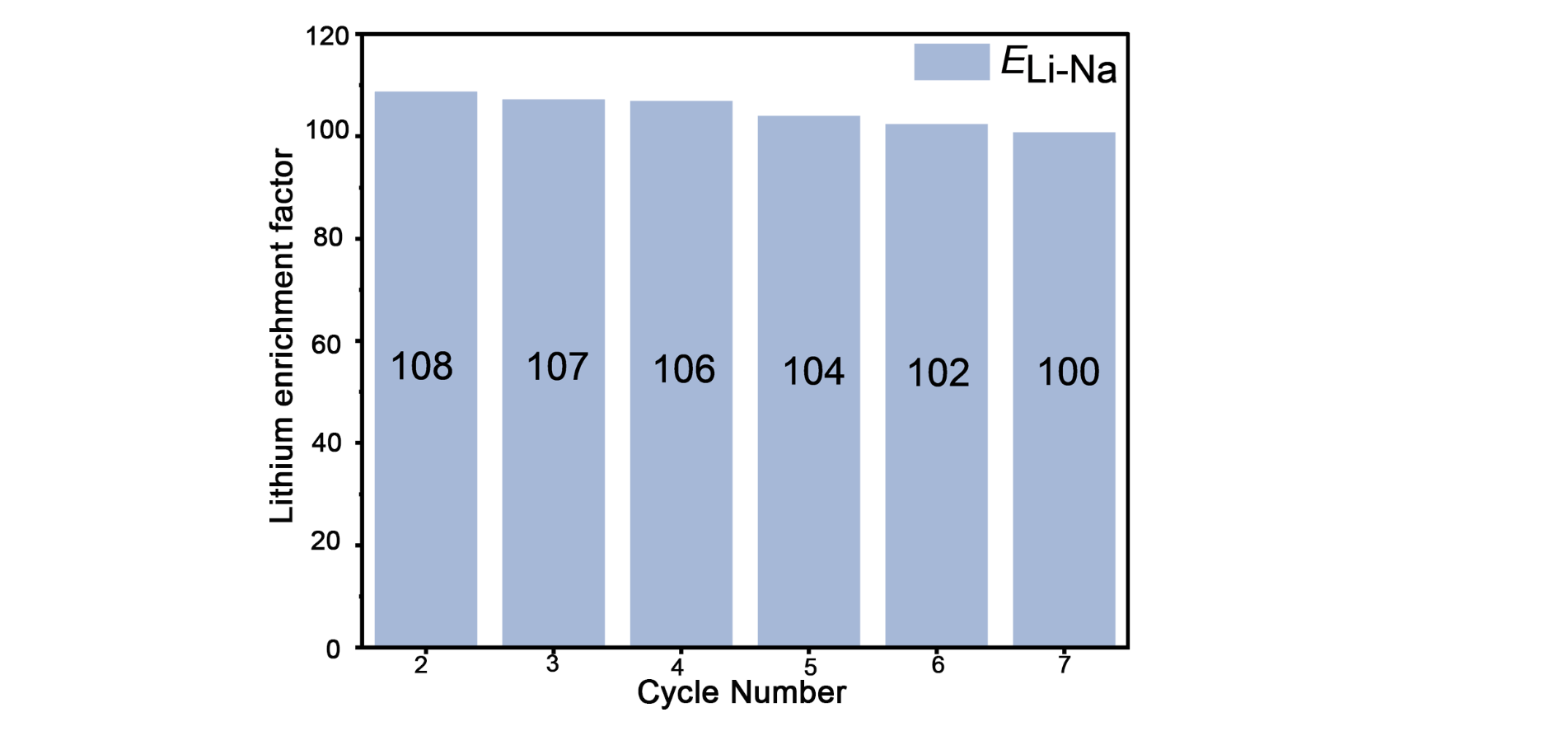


**Figure S9.** Lithium enrichment factor during cycles


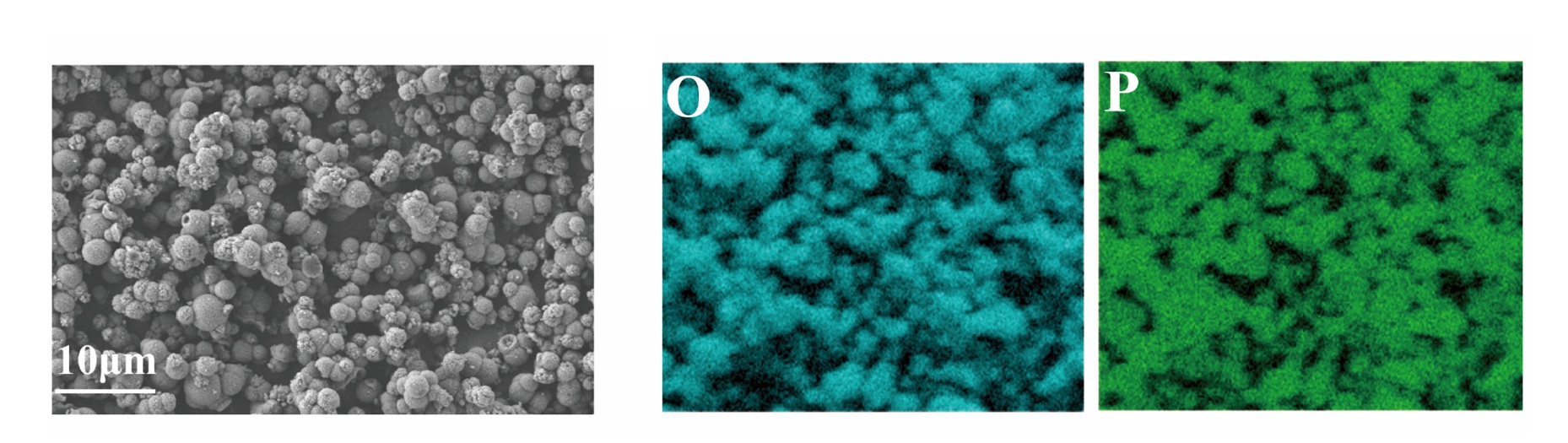


**Figure S10.** SEM image and EDS mapping images of Li_3_PO_4_ product

**Table S1.** Thermodynamic data of components in the Li-Fe-P-H_2_O systems at 298.15K

| **Species** | | ***Δ_f_G_T_*^o^ (kJ·mol^–1^)** | **References** |
| --- | --- | --- | --- |
| Li^+^ | –292.61 | | ^[1]^ |
| Fe^2+^ | –78.75 | | ^[1]^ |
| Fe^3+^ | –4.54 | | ^[1]^ |
| Fe(OH)_2_ | –492.58 | | ^[1]^ |
| Fe(OH)_3_ | –683.83 | | ^[1]^ |
| H_3_PO_4_ | –1148.02 | | ^[1]^ |
| H_2_PO_4_^–^ | –1135.77 | | ^[1]^ |
| HPO_4_^2–^ | –1094.71 | | ^[1]^ |
| PO_4_^3–^ | –1005.90 | | ^[1]^ |
| FePO_4_·2H_2_O | –1645.48 | | ^[1]^ |
| LiFePO_4_ | –1517.7 | | ^[1]^ |
| H_2_O | –237.1 | | ^[1]^ |
| O_2_ | 0.00 | | ^[2]^ |
| H_2_ | 0.00 | | ^[2]^ |

**Table S2.** E-pH formulas of equilibrium reaction in the Li-Fe-P-H_2_O systems at 298.15 K

| **No.** | **Reaction formula** | ***E*-pH formula** |
| --- | --- | --- |
| 1 | 2H^+^+2e^-^=H_2_ | *E*=-0.0592pH |
| 2 | O_2_+4H^+^+4e^-^=2H_2_O | *E*=1.229-0.0592pH |
| 3 | Fe^3+^+e^-^=Fe^2+^ | *E*=0.769-0.0592lg[Fe^2+^]+0.0592lg[Fe^3+^] |
| 4 | Fe^3+^+H_3_PO_4_+2H_2_O=FePO_4_·2H_2_O+3H^+^ | pH=-1.082-1/3lg[Fe^3+^]-1/3lg[H_3_PO_4_] |
| 5 | FePO_4_·2H_2_O+3H^+^+e^-^=Fe^2+^+H_3_PO_4_+2H_2_O | *E*=0.578-0.0592lg[Fe^2+^]-0.0592lg[Fe^3+^]-0.177pH |
| 6 | FePO_4_·2H_2_O+Li^+^+e^-^=LiFePO_4_+2H_2_O | *E*=0.56+0.0592lg[Li^+^] |
| 7 | FePO_4_·2H_2_O+H_2_O+3Li^+^=Fe(OH)_3_+Li_3_PO_4_+3H^+^ | pH=7.214-lg[Li^+^] |
| 8 | Fe^2+^+H_3_PO_4_+Li^+^=LiFePO_4_+3H^+^ | pH=0.098-1/3lg[Li^+^]-1/3lg[Fe^2+^]-1/3lg[H_3_PO_4_] |
| 9 | LiFePO_4_+2H_2_O+2Li^+^=Fe(OH)_2_+Li_3_PO_4_+2H^+^ | pH=11.5368-lg[Li^+^] |
| 10 | Fe(OH)_4_^-^+Li_3_PO_4_+4H^+^+e^-^=LiFePO_4_+2Li^+^+4H_2_O | *E*=2.628-0.118lg[Li^+^]-0.237pH+0.05921lg[Fe(OH)_4_^-^] |
| 11 | Fe(OH)_3_+Li_3_PO_4_+3H^+^+e^-^=LiFePO_4_+2Li^+^+3H_2_O | *E*=1.8403-0.1183lg[Li^+^]-0.17738pH |
| 12 | Fe(OH)_4_^-^+H^+^=Fe(OH)_3_+H_2_O | pH=13.3165+lg[Fe(OH)_4_^-^] |
| 13 | Fe(OH)_4_^-^+e^-^+2H^+^=Fe(OH)_2_+2H_2_O | *E*=1.2634-0.1183pH+0.0592lg[Fe(OH)_4_^-^] |
| 14 | Fe(OH)_3_+H^+^+e^-^=Fe(OH)_2_+H_2_O | *E*=0.476-0.0592pH |
| 15 | H_2_PO_4_^-^+H^+^=H_3_PO_4_ | pH=2.146+lg[H_2_PO_4_^-^] |
| 16 | HPO_4_^2-^+H^+^=H_2_PO_4_^-^ | pH=7.194-lg[H_2_PO_4_^-^]+lg[HPO_4_^2-^] |
| 17 | PO_4_^3-^+H^+^=HPO_4_^2-^ | pH=15.556-lg[HPO_4_^2-^]+lg[PO_4_^3-^] |
| 18 | Li_3_PO_4_+2H^+^=3Li^+^+H_2_PO_4_ | pH=5.305-1.5lg[Li^+^] |
| 19 | Fe^2+^+H_3_PO_4_+8H_2_O=Fe_3_(PO_4_)_2_·8H_2_O+6H^+^ | pH=1.536-1/3lg[H_3_PO_4_]-0.5lg[Fe^2+^] |
| 20 | 3FePO_4_·2H_2_O+2H_2_O+2H^+^+3e^-^=Fe_3_(PO_4_)_2_·8H_2_O+H_2_PO_4_^-^ | *E*=0.353-0.0394pH-0.0197lg[H_2_PO_4_^-^] |
| 21 | 3FePO_4_·2H_2_O+2H_2_O+H^+^+3e^-^=Fe_3_(PO_4_)_2_·8H_2_O+HPO_4_^2-^ | *E*=0.211-0.0197pH-0.0197lg[HPO_4_^2-^] |
| 22 | Fe_3_(PO_4_)_2_·8H_2_O=3Fe(OH)_2_+2HPO_4_^2-^+4H^+^+2H_2_O | pH=10.320+0.5lg[HPO_4_^2-^] |
| 23 | 3Fe(OH)_3_+2HPO_4_^2-^+7H^+^+3e^-^=Fe_3_(PO_4_)_2_·8H_2_O+H_2_O | *E*=1.290-0.138pH+0.0394lg[HPO_4_^2-^] |
| 24 | 3Fe^2+^+2H_2_PO_4_^-^+8H_2_O=Fe_3_(PO_4_)_2_·8H_2_O+4H^+^ | pH=1.2317-0.75lg[Fe^2+^]-0.5lg[H_2_PO_4_^-^] |
| 25 | 3FePO_4_·2H_2_O+2H_2_O+3e^-^=Fe_3_(PO_4_)_2_·8H_2_O+PO_4_^3-^ | *E*=-0.0479-lg[PO_4_^3-^] |
| 26 | Fe^2+^+H_3_PO_4_+8H_2_O=Fe_3_(PO_4_)_2_·8H_2_O+6H^+^ | pH=1.536-1/3lg[H_3_PO_4_]-0.5lg[Fe^2+^] |

**Table S3.** ICP analysis of the materials after LSV

| Contents  Samples | Li/ppm | Fe/ppm | P/ppm | Na/ppm |
| --- | --- | --- | --- | --- |
| Li_0.52_FePO_4_ | 5.58 | 82.61 | 49.54 | 6.35 |
| Li_0.38_FePO_4_ | 2.05 | 27.36 | 14.16 | 2.8 |
| Li_0.19_FePO_4_ | 1.92 | 26.79 | 14.05 | 2.14 |
| FePO_4_ | 0.260.70 | 36.12 | 19.03 | 3.86 |

**Table S4.** The composition of the SLB-lepidolite before/after lithium extraction reaction

| **Component** | **Li^+^**  **/g·L^-1^** | **Na^+^**  **/g·L^-1^** | **K^+^**  **/g·L^-1^** | **Mg^2+^**  **/g·L^-1^** | **Ca^2+^**  **/g·L^-1^** | **SO_4_^2-^/g·L^-1^** |
| --- | --- | --- | --- | --- | --- | --- |
| Before | 1.07 | 21.69 | 10.00 | 0.15 | 0.28 | 65.40 |
| After | 0.02 | 70.25 | 10.32 | 0.13 | 0.27 | 187.16 |

**Table S5.** The composition of SLB-batteries before/after lithium extraction reaction

| **Component** | **Li^+^/g·L^-1^** | **Na^+^/ g·L^-1^** | **SO_4_^2-^/g·L^-1^** |
| --- | --- | --- | --- |
| Before | 2.10 | 49.50 | 117.70 |
| After | 0.2 | 87.40 | 183.77 |

**Table S6.** The composition of NLB-Zabuye before/after lithium extraction reaction

| **Component** | **Li^+^/ g·L^-1^** | **B/ g·L^-1^** | **K/g·L^-1^** | **Na^+^/ g·L^-1^** | **SO_4_^2-^/g·L^-1^** |
| --- | --- | --- | --- | --- | --- |
| Before | 0.698 | 2.53 | 14.8 | 84.5 | 14.13 |
| After | 0.056 | 2.51 | 14.63 | 94.2 | 15.02 |

**Table S7.** The composition of SLB and NLB solutions

| **Brine** | **Li^+^/ g·L^-1^** | **Na^+^/ g·L^-1^** | **K^+^/g·L^-1^** | **Mg^2+^/ g·L^-1^** | **Ca^2+^/g·L^-1^** |
| --- | --- | --- | --- | --- | --- |
| SLB-spodumene | 2.10 | 49.55 | - | - | - |
| SLB-lepidolite | 1.07 | 21.69 | 10.00 | 0.15 | 0.28 |
| SLB-batteries | 2.10 | 49.50 | - | - | - |
| NLB-Zabuye | 0.698 | 84.5 | 14.8 | 116.0 | - |
| NLB-Atacama | 1.428 | 75.05 | 18 | 9.86 | 0.20 |

**Table S8.** Comparison of lithium extraction performance between Li_0.19_FePO_4_ and representative materials reported in recent literature

| Material | Method Type | Capacity  (mg /g) | Selectivity  (α_Li-Na_) | Cycle Stability | Ref. |
| --- | --- | --- | --- | --- | --- |
| **Li_0.19_FePO_4_** | **Chemical Redox** | **33.22** | **1568** | **High Stable** | **This work (SLB-spodumene)** |
| FePO_4_ | Chemical Redox | 26.4 | 380 | Stable | ^[3]^ |
| Li_(4C−40%)_FePO_4_ | Electrochemical | 41.16 | 519 | 99.9% (10 cycles) | ^[4]^ |
| LiFePO_4_/FePO_4_ | Electrochemical | 33.78 | 103.4 | 80% (83 cycles) | ^[5]^ |
| Spent LiFePO_4_ | Electrochemical | 14.62 | 210.5 | ~60% (13 cycles) | ^[6]^ |
| Li_1−x_Ni_0.33_Co_1/3_Mn_1/3_O_2_/Ag | Electrochemical | 10.8 | 769 | Stable (20 cycles) | ^[7]^ |
| λ−MnO_2_ | Electrochemical | 13.9 | 38.78 | 82.8% (50 cycles) | ^[8]^ |
| PANI/LiMn_2_O_4_ | Electrochemical | 28.5 | - | 70.8%(200 cycles) | ^[9]^ |
| HMn_2_O_4_ | Chemical Adsorption | - | 27.2 | 85.4% (10 cycles) | ^[10]^ |
| LiFePO_4_ | Electrochemical | 17.1 | 100.63 | Stable (8 cycles) | ^[11]^ |

**Table S9.** Detailed evolution of ion concentrations during Step 1 and Step 2 processes over 7 cycles

| Cycle | Step 1: Lithium enrichment via delithiation  of SLFP | | | | Step 2: Lithium extraction from the salt lake brine using Li_0.19_FePO_4_ | | | |
| --- | --- | --- | --- | --- | --- | --- | --- | --- |
|  | $\text{c}_{\text{Li}^{\text{+}}}$  (g/L) | $\text{c}_{\text{Na}^{\text{+}}}$  (g/L) | $\text{c}_{\text{Fe}^{\text{3+}}}$  (g/L) | $\text{c}_{\text{p}}$  (g/L) | $\text{c}\text{'}_{\text{L}\text{i}^{\text{+}}}$  (g/L) | $\text{c}\text{'}_{\text{N}\text{a}^{\text{+}}}$  (g/L) | $\text{c}\text{'}_{\text{F}\text{e}^{\text{3+}}}$  (g/L) | $\text{c}\text{'}_{\text{P}}$  (g/L) |
| 1 | 2.695 | - | 0.067 | 0.021 | 0.040 | 45.0 | 0.02 | 0.150 |
| 2 | 3.725 | 0.375 | 0.01 | 0.021 | 0.040 | 48.3 | 0.01 | 0.086 |
| 3 | 4.570 | 0.571 | 0.023 | 0.042 | 0.038 | 48.3 | 0.01 | 0.086 |
| 4 | 4.902 | 0.626 | 0.028 | 0.034 | 0.031 | 46.8 | 0.031 | 0.024 |
| 5 | 5.250 | 0.691 | 0.042 | 0.033 | 0.037 | 44.2 | 0.036 | 0.030 |
| 6 | 5.605 | 0.724 | 0.048 | 0.033 | 0.040 | 48.6 | 0.036 | 0.030 |
| 7 | 5.827 | 0.845 | 0.056 | 0.035 | 0.031 | 45.6 | 0.031 | 0.021 |

Notes: $\text{c}_{\text{L}\text{i}^{\text{+}}}$, $\text{c}_{\text{N}\text{a}^{\text{+}}}$, $\text{c}_{\text{F}\text{e}^{\text{3+}}}$, and $\text{c}_{\text{P}}$ denote the mass concentrations of the respective ions in the aqueous solution during the Step 1 lithium enrichment process. Correspondingly, $\text{c}\text{'}_{\text{L}\text{i}^{\text{+}}}$, $\text{c}\text{'}_{\text{N}\text{a}^{\text{+}}}$, $\text{c}\text{'}_{\text{F}\text{e}^{\text{3+}}}$, and $\text{c}\text{'}_{\text{P}}$ represent the mass concentrations of the corresponding ions in the salt lake brine during the Step 2 lithium extraction process.

**Table S10.** Preprocessing throughput information

| **Preprocessing throughput** | | | |
| --- | --- | --- | --- |
|  | **Type** | **tonne/yr** | **Geographic location** |
| SLFP | Manufacturing scrap: electrode | 10,000 | China |

**Table S11.** Basic and original data of techno-economic analysis

| No. | Item | Market  price | Unit | Data Sources |
| --- | --- | --- | --- | --- |
| 1 | Spent LFP | 8200 | CNY/t | SMM |
| 2 | H_2_O_2_(27.5%) | 1800 | CNY/t | 100PPI |
| 3 | H_2_SO_4_(98%) | 600 | CNY/t | 100PPI |
| 4 | Na_2_SO_3_ | 2700 | CNY/t | 100PPI |
| 5 | Na_3_PO_4_·12H_2_O | 2300 | CNY/t | 100PPI |
| 6 | NaOH | 851 | CNY/t | 100PPI |
| 7 | Li_3_PO_4_ | 75000 | CNY/t | 100PPI |
| 8 | Water | 4.40 | CNY/t | HNZWFW |
| 9 | LiFePO_4_ cathode  material | 33740 | CNY/t | SMM |
| a) 1 $ = 7.2095 CNY (Update time: 2025/05/20);  b) Data sources: SMM (<https://new-energy.smm.cn/new_energy/14042>), 100PPI(<http://www.100ppi.com/ppi/>),HNZWFW(<https://zz.hnzwfw.gov.cn/>) | | | | |

**Table S12.** Recycling cost ($ per kg feedstock) of different battery recycling technologies

|  | **Pyro** | **Hydro** | **Regeneration** | **This work** |
| --- | --- | --- | --- | --- |
| Materials | 0.26 | 0.65 | 0.47 | 0.53 |
| Utilities | 0.11 | 0.06 | 0.19 | 0.05 |
| Other variable costs | 0.01 | 0.02 | 0.01 | 0.02 |
| Labor | 0.03 | 0.07 | 0.07 | 0.07 |
| Maintenance | 0.18 | 0.12 | 0.13 | 0.11 |
| Plant overhead | 0.05 | 0.08 | 0.12 | 0.14 |
| Other fixed costs | 0.23 | 0.16 | 0.18 | 0.15 |
| Annualized capital cost | 1.08 | 0.71 | 0.80 | 0.67 |
| Feedstock payment | (2.00) | (2.00) | (2.00) | (2.00) |

**Table S13.** Recycling profit ($ per kg feedstock) of different battery recycling technologies

|  | **Pyro** | **Hydro** | **Regeneration** | **This work** |
| --- | --- | --- | --- | --- |
| Cost | 0.03 | 0.15 | 0.15 | 0.26 |
| Revenue | 1.25 | 2.08 | 5.23 | 7.49 |
| Profit | 1.15 | 1.94 | 5.20 | 7.23 |

**Table S14.** Life-cycle environmental impacts of different recycling methods

|  | **Pyro** | **Hydro** | **Regeneration** | **This work** |
| --- | --- | --- | --- | --- |
| **Total energy use in MJ per kg cell recycled** | | | | |
| Total Energy | 19.980 | 24.046 | 8.732 | 11.884 |
| Fossil fuels | 17.145 | 22.303 | 7.792 | 11.438 |
| Coal | 10.101 | 6.234 | 6.664 | 3.702 |
| Natural gas | 6.002 | 1793.027 | 3.146 | 9.690 |
| Petroleum | 1.042 | 4.041 | 3.1821.15 | 3.247 |
| Water consumption (gal/kg) | 1.5 | 2.5 | 1.4 | 2.2 |
| **Total Emissions in g per kg cell recycled** | | | | |
| VOC | 0.181 | 0.329 | 0.117 | 0.165 |
| CO | 0.718 | 1.153 | 0.483 | 0.623 |
| NO_x_ | 1.469 | 2.146 | 1.105 | 1.252 |
| PM10 | 0.195 | 0.161 | 0.104 | 0.076 |
| PM2.5 | 0.105 | 0.121 | 0.068 | 0.064 |
| SO_x_ | 1.086 | 2.163 | 0.438 | 0.672 |
| BC | 0.017 | 0.031 | 0.023 | 0.025 |
| OC | 0.017 | 0.036 | 0.013 | 0.018 |
| CH_4_ | 2.718 | 3.929 | 1.196 | 1.982 |
| N_2_O | 0.033 | 0.036 | 0.016 | 0.019 |
| CO_2_ | 2395 | 1464 | 794 | 736 |
| CO_2_ (w/C in VOC&CO) | 2396 | 1467 | 795 | 738 |
| GHGs | 2486 | 1594 | 835 | 802 |

Note: The unit price is obtained from EverBatt 2023 database.

**Table S15.** Comparison the technical and economic index of different methods

| Methods | Lithium Recovery  % | Water consumption (m^3^) | Profit  ($ kg^-1^) | References |
| --- | --- | --- | --- | --- |
| **Cascade utilization** | **99** | **8.6×10^-4^** | **7.23** | **This work** |
| Hydrometallurgical method | 99.9 | 1.5×10^-3^ | 0.51 | ^[12]^ |
| Hydrometallurgical method/Regeneration | 97.6 | / | 0.295 | ^[13]^ |
| Hydrometallurgical method | 96.3 | 1.9×10^-2^ | 1.556 | ^[14]^ |
| Hydrometallurgical method | 94.1 | / | 0.647 | ^[15]^ |
| Regeneration-solid-phase method | / | / | / | ^[16]^ |
| Direct regeneration | / | / | 1.833 | ^[17]^ |
| Direct regeneration | / | / | 3.39 | ^[18]^ |
| Direct regeneration | / | 0.2 | 1.28 | ^[19]^ |

**Text S1.** Computational details

All calculations were carried out for the material in the framework of Density Functional Theory (DFT) using the Vienna Ab initio Simulation Package (VASP 6.3.2). The generalised gradient approximation (GGA) of the Perdew-Burke-Ernzerhof (PBE) function was used to describe the exchange-correlation energy. The projected augmented wave (PAW) method and pseudopotentials were used to describe the interactions between valence electrons and ions. To ensure the efficiency of the computational results and parallel computing. A 3*3*3 k-point grid under Monkhorst-Pack is used in the optimisation process and 500 eV truncation energy is set. The lattice parameters and ionic positions of all crystals were fully relaxed, and the convergence criteria for the total energy of all relaxed atoms and the final force were 10^-6^ eV and 0.03 eV/Å, respectively. To correct the localization effect of electrons on the d orbital of transition metal atoms, the Hubbard+U method is adopted, where the effective U value (Ueff) of Fe is 2.5eV.

**Text S2.** Thermodynamic Calculation of Redox Potentials

To validate the thermodynamic feasibility of the process in the complex brine environment, the redox potentials of the reductant (SO_3_^2-^/SO_4_^2-^) and the oxidant (FePO_4_/LiFePO_4_) were calculated based on the Nernst equation.

The thermodynamic potential of the SO_3_^2-^/SO_4_^2-^ reductant is determined by its oxidation half-reaction in an alkaline medium, which is expressed as follows:

$$\text{S}\text{O}_{\text{4}}^{\text{2-}}\text{+}\text{H}_{\text{2}}\text{O+2}\text{e}^{\text{-}}\text{⇌}\text{S}\text{O}_{\text{3}}^{\text{2-}}\text{+2O}\text{H}^{\text{-}}\text{ }\left( \text{E}^{\text{0}}\text{≈-0.93 V vs. SHE} \right)$$

According to the Nernst equation:

$$\text{E}_{\text{SO}_{\text{3}}^{\text{2-}}\text{-}\text{/}\text{SO}_{\text{4}}^{\text{2-}}}\text{=}\text{E}^{\text{0}}\text{-}\frac{\text{RT}}{\text{nF}}\text{ln}\left( \frac{\left[ \text{S}\text{O}_{\text{3}}^{\text{2-}} \right]\text{⋅}\left[ \text{O}\text{H}^{\text{-}} \right]^{\text{2}}}{\left[ \text{S}\text{O}_{\text{4}}^{\text{2-}} \right]} \right)$$

$$\text{E}_{\text{SO}_{\text{3}}^{\text{2-}}\text{-}\text{/}\text{SO}_{\text{4}}^{\text{2-}}}\text{=-0.93-0.0295lg}\left( \frac{\left[ \text{S}\text{O}_{\text{3}}^{\text{2-}} \right]}{\left[ \text{S}\text{O}_{\text{4}}^{\text{2-}} \right]} \right)\text{-0.0591×}\left( \text{14-pH} \right)$$

To evaluate the thermodynamic feasibility under the limiting conditions at the reaction endpoint (pH 7.2), we assumed the reductant was nearly depleted with a concentration ratio of $\left[ \text{S}\text{O}_{\text{3}}^{\text{2-}} \right]\text{/}\left[ \text{S}\text{O}_{\text{4}}^{\text{2-}} \right]\text{≈}\text{10}^{\text{-6}}$. Substituting these values into the Nernst equation yields a theoretical anode potential of approximately $\text{-0.35 V (vs. SHE).}$

$$\text{E}_{\text{SO}_{\text{3}}^{\text{2-}}\text{-}\text{/}\text{SO}_{\text{4}}^{\text{2-}}}\text{≈-0.93-0.0295×}\left( \text{-6} \right)\text{-0.0591×}\left( \text{6.8} \right)\text{≈-0.35 V (vs. SHE)}$$

However, considering the high ionic strength of the brine which may affect the activity coefficients of ions, we adopted a more conservative value of -0.2 V (vs. SHE) as the upper threshold for the potential. Concurrently, regarding the oxidant ($\text{FeP}\text{O}_{\text{4}}\text{/}\text{LiFeP}\text{O}_{\text{4}}$), the standard potential corresponds to $\text{≈0.41}$ V vs. SHE, while in the specific brine environment, taking into account the open-circuit potential (OCP) stabilization, the plateau is approximately 0.43 V (vs. SHE). Consequently, even under these most unfavorable conditions, the potential difference ($\text{ΔE=}\text{E}_{{\text{LiFePO}_{\text{4}}\text{-FePO}}_{\text{4}}}\text{-}\text{E}_{\text{SO}_{\text{3}}^{\text{2-}}\text{-}\text{/}\text{SO}_{\text{4}}^{\text{2-}}}\text{≈0.43-}\left( \text{-0.2} \right)\text{=0.63V).}$ Since $\text{ΔE>0}$, the Gibbs free energy change $\text{ΔG=-nFΔE}\text{≪}\text{0}$, confirming that the reaction remains thermodynamically spontaneous throughout the entire process.

**Text S3.** Economic Evaluation Details

A laboratory-scale economic evaluation of the recycling of spent LiFePO_4_ (SLFP) was performed based on the proposed recycling process using 1 kg of SLFP as an example. The cost evaluation of the integrated design process was shown in Table S11. The details were as follows (1$ = 7.2095 RMB). The whole recycling process of the SLFP could be divided into three parts: (i) leaching process; (ii) lithium extraction from secondary-treatment lithium-containing brine-battery; (iii) Li_3_PO_4_ product.

(i) Leaching process

The input cost of 1 kg of used LFP is 1.60 $. The prices of H_2_O_2_ (27.5%) and H_2_SO_4_ are 0.25 $/kg (27.5% H_2_O_2_) and 0.08 $/kg (98% H_2_SO_4_), respectively. The oxidative leaching process followed the chemical reaction 2LiFePO_4_ + H_2_SO_4_+ H_2_O_2_= Li_2_SO_4_ + 2FePO_4_ +2H_2_O, in which a 25% excess of hydrogen peroxide was required.

(ii) Lithium extraction from secondary-treatment lithium-containing brine-battery

The lithium recovery process was carried out on a laboratory scale. The first step of the leaching process yields approximately 0.95 kg of Li_0.19_FePO_4_ from 1 kg of spent LFP due to losses during the experiment. The reduction reaction followed the equation Fe^3^⁺+SO_3_^2−^ + 2OH^−^ − e^−^ = SO_4_^2−^ + Fe^2^⁺ +H_2_O The equation, in which 25% Na_2_SO_3_ was required.

(iii) Li_3_PO_4_ product

In order to remove impurities such as Fe ions from the leach solution, NaOH was added, and the reaction follows Na_3_PO_4_+ Li_2_SO_4_ = Li_3_PO_4_+ Na_2_SO_4_ with the market price of NaOH (98%) and Na_3_PO_4_·12H_2_O (98%) at $0.11/kg and $0.31/kg, respectively.

Under ideal conditions, the inputs and outputs for each stage were estimated based on a material balance. Actual industrial applications still need to consider labor and operating costs as well as equipment losses.

**Text S4.** Materials

The H_2_O_2,_ H_2_SO_4,_ Na_2_SO_3_, Na_3_PO_4_⋅12H_2_O and N-methyl pyrrolidone (NMP) used were all analytical grade reagents, and they were all sourced from Sinopharm Chemical Reagent Co., Ltd. The polyvinylidene fluoride (PVDF) and acetylene black were purchased from Shanghai Fukang New Material Technology Co., Ltd and Tianjin Huaran Chemical Co., Ltd, respectively. The SLFP is supplied by Hunan Shunhualithium Co., Ltd., which is a trimmed piece from the coating machine. The deionized water was self-made by the laboratory.

**Text S5.** Material characterization

The material phase identification was carried out using X-ray diffractometer (XRD, Rigaku-SmartLab SE, Japan). The morphology of the materials was analyzed using a scanning electron microscope (SEM, TESCAN, MIR3 LMH, Czechia). The distribution of elements is analyzed using energy-dispersive X-ray spectrometry (EDS). An electron probe microanalyzer (EPMA, Shimadzu Corporation, Japan) was carried out to conduct phase analysis of the micro-region chemical composition of the substance and observe the micro-region morphology. X-ray photoelectron spectroscopy (XPS, Thermo Fisher Scientific-K-Alpha, USA) was utilized to analyze the valence state content of elements in the material. The analysis of elemental contents was performed by inductively coupled plasma optical emission spectrometer (ICP, Agilent 5800VDV, USA). Fourier transform infrared spectrometer (FT-IR, BRUKER INVENIO S, Germany) was adopted to study the structure and composition of substance molecules. Raman spectroscopy (Raman, Thermo Fisher-Dxr 3xi, USA) was employed to obtain information on molecular vibration and rotation to analyze the molecular structure. Physical particle abundance was measured and analyzed using a laser particle size analyzer (Microtrac SYNC, USA).

**Text S****6.** Data analysis

(1) Leaching rate

During the delithiation process, the leaching rate can be calculated by equation 2-1:

|  | $\text{X}_{\text{i}}\text{=}\frac{\text{c}_{\text{i}}\text{×}\text{V}}{\text{m}\text{×}\text{ω}_{\text{i}}}\text{×100\%}$ | (2-1) |
| --- | --- | --- |

Among them: *c_i_* (g·L^-1^) is the concentration of element i in the solution measured by ICP-OES; *V* (L) is the volume of the reaction solution; *m* (g) and*ω_i_* refer to the mass of the raw material SLFP and the mass fraction of element i.

(2) Recovery rate/precipitation rate

The recovery rate of Li^+^ during lithium intercalation or the precipitation rate of Li^+^ during the preparation of Li_3_PO_4_ can be calculated by equation 2-2:

|  | $\text{Y}_{\text{Li}}\text{=}\frac{\text{c}_{\text{MLi}}\text{-}\text{c}_{\text{Li}}}{\text{C}_{\text{MLi}}}\text{×100\%}$ | (2-2) |
| --- | --- | --- |

Where $\text{c}_{\text{MLi}}$ (g·L^-1^) is the concentration of Li^+^ in brine solution or oxidation leaching solution; and $\text{c}_{\text{Li}}$ (g·L^-1^) is the concentration of Li^+^ in the solution after the reaction is complete.

(3) Lithium enrichment factor

The lithium enrichment factor in the lithium extraction process is calculated by 2-3:

|  | $\text{E}_{\text{L}\text{i-Na}}\text{=}\frac{\text{(}\text{c}_{\text{Li}}\text{/}\text{c}_{\text{Na}}\text{)}\text{ }_{\text{t}}}{\text{c}_{\text{Li0}}\text{/}\text{c}_{\text{Na0}}}$ | (2-3) |
| --- | --- | --- |

where $\text{E}$_Li-Na_ represents the separation coefficient of Li^+^ and Na^+^ ions; $\text{c}_{\text{Li0}}$ and $\text{c}_{\text{Na0}}$ represent the concentration of Li^+^ and Na^+^ in the brine (g⋅L^-1^). $\text{c}_{\text{Li}}$ and $\text{c}_{\text{Na}}$ refer to the concentration of Li and Na during oxidation (g⋅L^-1^). t represents the number of cycles.

(4) Mass fraction

The mass fraction of each atom of synthesized Li_3_PO_4_ can be calculated by equation 2-4:

|  | $\text{W}_{\text{i}}\text{=}\frac{\text{c}_{\text{i}}\text{*}\text{10}^{\text{-3}}\text{*}\text{D}}{\text{M}}\text{×100\%}$ | (2-4) |
| --- | --- | --- |

In this case: $\text{c}_{\text{i}}$ (g·L^-1^) is the concentration of element i in solution as determined by ICP-OES; *D* is the dilution factor, and *M* (g) is the mass of the sample dissolved in the aqua regia.

(5) Chemical Oxygen Demand (COD)

|  | *ρ*(COD_Cr_)=*c*(*V_0_*-*V_1_*)$\text{×}$8000/*V_2_* | (2-5) |
| --- | --- | --- |

The COD value is determined using the dichromate titration method as specified in HJ828-2017. *c* is the concentration of the standard ammonium ferric sulfate solution at 0.0962 mol/L. *V_2_* is the volume of the sample, which is 10 mL. *V_0_* is the initial volume of 25.093 mL. *V_1_* is the volume of potassium dichromate solution added during the experiment.

(6) Adsorption capacity/Lithium extraction capacity

The lithium adsorption capacity/Lithium extraction capacity, denoted as Q_e_ (mg/g), is defined as the mass of lithium ions extracted from the brine per unit mass of the adsorbent. It is calculated based on the concentration difference of Li^+^ in the solution before and after the reaction, according to equation 2-6:

|  | $\text{Q}_{\text{e}}\text{=}\frac{{\text{(}\text{c}}_{\text{Li}}\text{-}\text{c}_{\text{e}}\text{)×}\text{V}}{\text{m}}$ | (2-6) |
| --- | --- | --- |

Where $Q$_e_ represents the adsorption capacity (mg/g); $\text{c}_{\text{Li}}$ is the initial concentration of lithium ions in the brine (mg/L); $\text{c}_{\text{e}}$ is the concentration of lithium ions in the supernatant after adsorption (mg/L). It is calculated by measuring the depletion of lithium ions in the liquid phase using ICP-OES. *V* is the volume of the brine solution (L); *m* is the mass of the adsorbent powder used (g).

(7) Separation coefficient

The separation coefficient of Li^+^ to Na^+^ in the lithium extraction process is calculated by 2-7:

|  | $\text{α}_{\text{Li-Na}}\text{=}\frac{\left( \text{c}_{\text{Li}}^{\text{0}}\text{/}\text{c}_{\text{Na}}^{\text{0}} \right)_{\text{s}}}{\left( \text{c}_{\text{Li}}\text{/}\text{c}_{\text{Na}} \right)_{\text{l}}}$ | (2-7) |
| --- | --- | --- |

Where $\alpha_{\text{L}\text{i-Na}}$represents the separation factor of Li^+^ over Na^+^.$\text{c}_{\text{Li}}^{\text{0}}$ and $\text{c}_{\text{Na}}^{\text{0}}$ are the concentration of Li^+^ and Na^+^ in the solid materials; and $\text{c}_{\text{Li}}$and $\text{c}_{\text{Na}}$ are the concentration of Li^+^ and Na^+^ in the brine (g⋅L^-1^).

**Text S7.** Electrochemical measurements

Mixing Li*_x_*FePO_4_ powder, PVDF binder, and acetylene black conductive agent in a mass ratio of 8:1:1, and preparing a slurry through NMP. The slurry was evenly coated on the titanium sheet with a 200 μm coating rod and then dried at 80 ℃ for 12 h. The linear sweep voltammetry (LSV), and chronopotentiometry (CP) tests were performed using a three-electrode system. Concretely, the obtained Li*_x_*FePO_4_ electrode, graphite sheet, and saturated calomel electrode (SCE) served as the working electrode, counter electrode, and reference electrode, respectively. Moreover, the surface of the working electrode and counter electrode were 1×1 cm^2^ and 4×4 cm^2^, respectively. Furthermore, the electrochemical tests were carried out in an electrochemical workstation (Shanghai Chenhua Electrochemical Workstation, CHI600E). The CV test was carried out in Li_2_SO_4_ solution. The LSV test and CP test were carried out in a mixture solution (Li^+^ 2.0 g⋅L^-1^, Na^+^ 55.0 g⋅L^-1^). The voltage scanning range of the CV and LSV test was -0.6-0.6 V (vs SCE), and the scanning rate was 0.25 mV·s^-1^. The current density of the CP test was 0.2 mA·m^−2^. The electrochemical properties of the SLFP and the recovered LiFePO_4_ were investigated using CR2032 coin cells. The electrode slurry was prepared by mixing the active material, acetylene black, and PVDF in NMP at a mass ratio of 8:1:1. The slurry was applied to Al foil and dried in a vacuum oven at 100 ℃ for over 14 h. Next, half-cells were assembled in an Ar-filled glove box (H_2_O < 0.1 ppm, O_2_ < 0.1 ppm) using lithium metal as the anode, a Celgard 2400 separator, and 1 M LiPF_6_ in EC:DEC:DMC (1:1:1 by volume) as the electrolyte. After standing for 12 h, the cells were tested on a LANHE CT2001A system at 25 ℃ within the voltage range of 2.5-4.3 V.

**References**

[1] C. Liu, *The Hand Book of Minerals and the Related Compounds Thermodynamic Data*, Science Press, Beijing **1985**.

[2] A. La Iglesia, J. F. Félix, *Geochim. Cosmochim. Acta* **1994**, *58* (19), 3983.

[3] J. Wang, A. W. Hawkins, A. T. Saasi, C. G. Morin, G. M. Geise, G. M. Koenig, *Sustainable Energy Fuels* **2024**, *8* (17), 3902.

[4] X. Zhao, S. Yang, X. Song, Y. Wang, H. Zhang, M. Li, Y. Wang, *Adv. Sci.* **2024**, *11* (41), 2405176.

[5] W. Zhu, W. Xu, D. Liu, L. He, Z. Zhao, *Desalination* **2025**, *614*, 119166.

[6] M. Du, J.-Z. Guo, S.-H. Zheng, Y. Liu, J.-L. Yang, K.-Y. Zhang, Z.-Y. Gu, X.-T. Wang, X.-L. Wu, *Chin. Chem. Lett.* **2023**, *34* (6), 107706.

[7] C. P. Lawagon, G. M. Nisola, R. A. I. Cuevas, H. Kim, S.-P. Lee, W.-J. Chung, *Chem. Eng. J.* **2018**, *348*, 1000.

[8] D.-F. Liu, S.-Y. Sun, J.-G. Yu, *Can. J. Chem. Eng.* **2019**, *97* (S1), 1589.

[9] A. Zhao, J. Liu, X. Ai, H. Yang, Y. Cao, *ChemSusChem* **2019**, *12* (7), 1361.

[10] L. Song, M. Liu, M. Nian, G. Yang, *RSC Adv.* **2024**, *14* (28), 19795.

[11] S. Sun, X. Yu, M. Li, J. Duo, Y. Guo, T. Deng, *J. Clean. Prod.* **2020**, *247*, 119178.

[12] J. Zhang, J. Hu, Y. Liu, Q. Jing, C. Yang, Y. Chen, C. Wang, *ACS Sustainable Chem. Eng.* **2019**, *7* (6), 5626.

[13] X. Qiu, B. Zhang, Y. Xu, J. Hu, W. Deng, G. Zou, H. Hou, Y. Yang, W. Sun, Y. Hu, X. Cao, X. Ji, *Green Chem.* **2022**, *24* (6), 2506.

[14] Y. Xu, X. Qiu, B. Zhang, A. Di, W. Deng, G. Zou, H. Hou, X. Ji, *Green Chem.* **2022**, *24* (19), 7448.

[15] Y. Yang, X. Meng, H. Cao, X. Lin, C. Liu, Y. Sun, Y. Zhang, Z. Sun, *Green Chem.* **2018**, *20* (13), 3121.

[16] L. Song, C. Qi, S. Wang, X. Zhu, T. Zhang, Y. Jin, M. Zhang, *Waste Manage.* **2023**, *157*, 141.

[17] X. Qiu, C. Wang, Y. Chen, Z. Du, L. Xie, Q. Han, L. Zhu, X. Cao, X. Ji, *Small* **2024**, *20* (40), 2402278.

[18] M. Xu, C. Wu, F. Zhang, Y. Zhang, J. Ren, C. Zhang, X. Wang, L. Xiao, O. Fontaine, J. Qian,*Energy Storage Mater.* **2024**, *71*, 103611.

[19] G. Ji, J. Wang, Z. Liang, K. Jia, J. Ma, Z. Zhuang, G. Zhou, H. M. Cheng, *Nat. Commun.* **2023**, *14* (1), 584.
